# *In Silico* identification of novel phytochemicals that target SFRP4: An early biomarker of diabesity

**DOI:** 10.1371/journal.pone.0292155

**Published:** 2023-11-09

**Authors:** Asim Rehman, Shazia Anwer Bukhari, Naheed Akhter, Muhammad Abdullah Ijaz Hussain, Zunera Chauhdary

**Affiliations:** 1 Department of Biochemistry, Government College University Faisalabad, Faisalabad, Pakistan; 2 Department of Chemistry, Government College University Faisalabad, Faisalabad, Pakistan; 3 Faculty of Pharmaceutical Sciences, Government College University Faisalabad, Faisalabad, Pakistan; The Islamia University of Bahawalpur Pakistan, PAKISTAN

## Abstract

The simultaneous coexistence of complicated metabolic conditions like obesity and diabetes within an individual is known as diabesity. Obesity is the key factor for many chronic diseases, including insulin resistance and type 2 diabetes (T2D). Metabolic stress due to nutrient overload releases different inflammatory mediators. Secreted frizzled-related protein 4 (SFRP4) is also an inflammatory mediator that impairs insulin secretion. SFRP4 acts as an early biomarker for diabesity expressed with interleukin-1 beta (IL-1β) in the adipose tissues that hinder the exocytosis of insulin-secreting granules from the pancreatic β-cells and is a potential target for preserving β-cell dysfunction and the diabesity treatment. The current study aimed to screen potential bioactive compounds targeting and inhibiting the diabesity-linked SFRP4 protein through an *in silico* approach. The three-dimensional (3D) structure of human SFRP4 was predicted through comparative modeling techniques and evaluated by various online bioinformatics tools. The molecular docking and MD simulation investigations were carried out against phytochemicals with anti-diabetic and anti-obesity properties to shortlist the best SFRP4 inhibitor. Hesperetin, Curcumin, Isorhamnetin, Embelin, Epicatechin, and Methyl Eugenol interacted strongly with SFRP4 by displaying zero RMSD and binding affinities of -6.5, -6.4, -6.3, -5.3, -6.3 and -5.8 kcal/mol respectively. Additionally, the root mean square fluctuation and root mean square deviation graphs from the MD simulation results demonstrated that hesperetin has good variations throughout the simulation period as compared to others. This dynamic stability and control behavior of hesperetin, when it interacts with SFRP4, shows that it has the potential to modulate the function and activity of the protein. Therefore, hesperetin is identified as an effective and top drug candidate through this analysis for preserving beta-cell function and treating diabesity by targeting SFRP4. The findings of this study could be useful in the design and development of diabesity drugs.

## Introduction

The term ‘diabesity’ was introduced by Sims and their colleagues in 1973 [1–4], to describe obesity-induced diabetes also referred to as "obesity-dependent diabetes [5]. Diabesity is the adverse health effect of type 2 diabetes mellitus (T2DM) and obesity. Diabesity is a metabolic disorder posing an immense economic burden on society and has arisen as a major health risk, quoting like a slow poison, matching with parallel affluence that cannot be controlled and cured with available remedies [3, 6]. There exists a strong link between obesity, insulin resistance, and the development of T2D [7]. The prevalence of T2D increases threefold and twentyfold with a body mass index (BMI) of 25 to 30 kg/m^2^ and above, respectively [8]. In particular, the accumulation of abdominal fat declines insulin secretion, hence obesity is a self-regulating and solid risk of developing T2D [9]. Insulin resistance and diabetes mellitus have a solid connection with obesity [10].

T2DM, also known as non-insulin-dependent diabetes, results in a defect in the insulin secretion pathway that leads to insufficient insulin secretion and production [11]. The Diabetes International Federation reported that at the end of 2017, 425 million people were diabetic, and the number of diabetic individuals will reach 628 million in 2045 [12]. The first and foremost cause of T2DM is related to secretory defects of insulin due to metabolic stress and inflammation, amongst other contributors like genetic factors. The risks of developing T2DM increase with age, overweight, and absence of physical activity. The excessive weight itself causes insulin resistance [13]. In obesity, increased concentrations of systemic pro-inflammatory cytokines and chemokines and down-regulation of anti-inflammatory adiponectin lead to long-lasting sub-clinical inflammation that is often linked with the development of insulin resistance, ß-cell dysfunction, and eventually T2DM [14].

In obesity, the tissues of adipose play a major role as an organ of the body in the development of diabetes. Adipose tissues are involved in the inflammatory process during obesity-induced T2DM and act as the main source of inflammatory mediators. Abdominal white adipose tissues play a critical role in inflammation and insulin resistance. White adipose tissues release different inflammatory mediators, like interleukin-6 and cytokines, that are associated with the inflammatory response [15].

An inflammation mediator called secreted frizzled-related protein 4 (SFRP4) is secreted by adipose tissues along with other inflammatory mediators like IL6, IL-1β, TNFα, and CRP. SFRP4 is secreted in the systemic circulation many years before the onset of diabetes and causes severe β-cell dysfunction, impaired glucose metabolism, and insulin resistance [16]. SFRP4 is found in both alpha and beta cells and is probably constitutively released from islets. SFRP4 is a 40-kilo Dalton protein having 346 amino acids with two domains. Number one is the amino terminus domain that shows signal peptide secretion and number two is the cysteine-rich domain with a structural resemblance to a frizzled (Fz) receptor and ended by a carboxy terminus [17]. SFRP4 is also the controller of the extracellular Wnt signaling (Wnt) pathway by binding with specific Wnt ligands via a cysteine-rich domain. Increased levels of SFRP4 in systemic circulation led to decreased glucose tolerance because of reduced expression of calcium channels in the islet thereby repressing exocytosis of insulin [18]. SFRP-4 is overexpressed in T2DM causing a decrease in insulin secretion and beta-cell dysfunction. SFRP4 could be a specific therapeutic target for treating islet dysfunction, and blocking SFRP-4 at an early stage increases insulin secretion [19].

Plant-based chemicals have acquired a lot of interest in treating diabetes and other disorders because of their low cost and lack of gastrointestinal, renal, or hypertensive adverse effects. In 75–80% of developing countries, plant-based compounds are used to treat a variety of diseases including diabetes and obesity [20]. Many plant-derived drugs have attained much importance for their safe use as medical therapy. In the past few years, the use of herbal medicines has increased. Researchers have investigated a variety of phytochemicals like terpenoids, tannins, flavonoids, alkaloids, steroids, saponins, glycosides, and proteins found in plants. These plant-derived chemical compounds and their products can be used as beneficial factors for the treatment of various metabolic disorders like obesity, cancer, and diabetes [21]. An ancient times to modern history, several traditional plant-based remedies are played a significant role in the health care system. An emerging discipline known as computer-aided drug design (CADD) is used as a central hub for potential drug discovery. CADD considerably reduces the cost of drug discovery and accelerates discovery time. A structure-based approach and a ligand-based approach are used in CADD. In the structure-based approach, the target’s structure is analyzed to identify key sites that are crucial for biological functions. In contrast, ligand-based approaches evaluate the activities and physiochemical properties of known ligands. It is a promising field that can be utilized in the design of novel drugs using computational techniques [22]. The methods of CADD include virtual screening, molecular docking, and molecular dynamics (MD) simulation. These approaches are the most preferred for analyzing and discovering drugs and biologically active compounds against a specific target [23]. These approaches allow us to acquire valuable insights into the behavior of the compounds, particularly regarding their interactions and the stability of their binding with biological targets. The current study is designed using in silico approaches to shortlist different phytochemicals with the potential to interact with the predicted 3D structure of SFRP-4 that can be further investigated *in vitro* and *in vivo* studies.

## Materials and methods

### Retrieval and analysis of sequence

**Fig 1:**
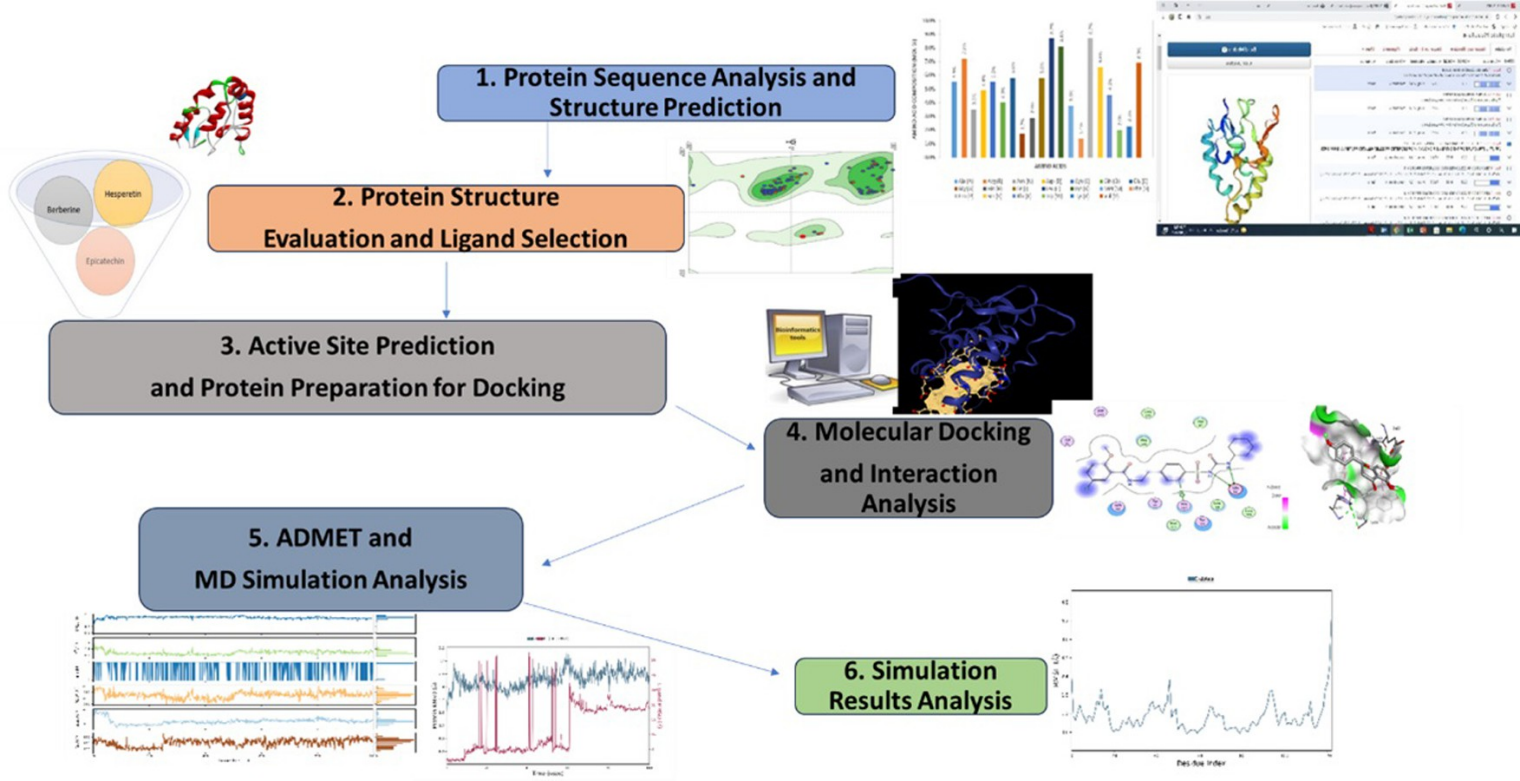
Schematic diagram of the study design.

The full-length amino acid sequence of homo sapiens SFRP4 in FASTA format was obtained from the protein database NCBI (https://www.ncbi.nlm.nih.gov/) using accession no. CAG46532.1. The physical properties, sequence analysis, and amino acid composition were done using ProtParam (https://web.expasy.org/cgi-bin/porparam/) (Fig 1) [24].

### 3D Structure prediction and evaluation

The SFRP4 protein 3D structure was predicted using Swiss homolog modeling (https://swissmodel.expasy.org/) [25]. Then the predicted model was refined by Galaxy Refine online software and the quality of the refined model was evaluated by different bioinformatics tools like proSA [26], PDBsum-EMBL [27], ERRAT, and Verify 3D [28].

### Ligands selection and preparation for docking

Plants with potential anti-diabetic and anti-obesity properties were chosen from already existing literature and the active phytochemicals were listed. A total of 300+ phytochemicals were retrieved from the Chemical Entities of Biological Interest (ChEBI) [29] and the PubChem [30] database (https://pubchem.ncbi.nlm.nih.gov/). The retrieved structure was saved in .sdf format. The compounds downloaded from the database were loaded onto PyRx docking software with the help of the Open Babel tool existing in the software. The energy minimization and force field optimization was done using uff geometry, and the optimization algorithm was set to conjugate gradients at a total 200 number of steps. The resulting optimized ligands were then changed and saved into the PDBQT format, a suitable input for docking by auto dock vina [31].

### Active site prediction and preparation of protein

The active pocket of the SFRP4 protein was predicted using the pocket analysis and detection tool DoGSiteScorer [32]. After manually examining the six predicted active pockets, we finally chose and proceeded with Active Pocket 1. The Swiss model PDB Viewer is used for the SFRP-4 protein energy minimization process. The energy-minimized structure of the protein in the PDB format was loaded onto PyRx, changed into the PDBQT format, and set as the target macromolecule ready for docking [33, 34].

### Molecular docking

Docking between SFRP4 protein and several phytochemical ligands was performed by virtual screening software PyRx using the AutoDock Vina run option [35]. The active site of protein predicted by using the DoGSiteScorer was set by using the grid box center and size dimensions. The grid box dimensions for the center were set at (x = 26.35, y = -9.37, and z = 57.33), and for the size were set at (x = 25.07 Å, y = 19.77 Å, and z = 25.0 Å) using default exhaustiveness value at 8 to maximize the protein-ligand binding conformational investigation [31].

### Study of ligands interactions

After docking, each phytochemical generated different poses of results. The pose with the maximum negative binding energy and zero RMSD value was selected [36]. The protein-ligand interaction results generated by autodock were observed and visualized with BIOVIA Discovery Studio [37, 38] and snaps were taken of the best interaction poses.

### ADME/T and biological properties analysis

The selected hit compounds after docking were screened based on Lipinski’s rule of five (RO5) [39] and ADME/T profiling which includes absorption, distribution, metabolism, excretion, and toxicity (ADME/Tox). All these drug-likeness properties were calculated and compounds toxicity was predicted by using computational tools like SwissADME [40], ADMETlab 2.0 [41], ProTox-II [42], pkCSM [43], and admetSAR [44]. The ligand’s SMILE format was collected from the Chemical entities of biological interest (ChEBI) [29] and the PubChem [30] database is used as input structures for all these above-mentioned servers.

### Molecular Dynamic (MD) simulation

The dynamic behavior and stability of the protein-ligand or docked complex under various conditions are frequently studied using the *in-silico* technique known as molecular dynamics (MD) simulation. Molecular docking studies in static situations may forecast a ligand’s binding state and give a static view of the molecule’s binding posture at the protein’s active site. Whereas, MD simulations utilize Newton’s classical equation of motion to track the movement of atoms across time and provide a dynamic perspective of molecular behavior. The Desmond/ Maestro (2022.1 version) was used to conduct simulation studies [45, 46]. In the Maestro interface, the receptor-ligand complexes were refined and preprocessed using the Protein Preparation Wizard, which included complex optimization and reduction. The system was set up using the System Builder tool. The dock complexes were solvated in an orthorhombic box, TIP3P (Transferable Intermolecular Interaction Potential 3 Points). The OPLS 2005 force field was utilized for the simulation and to balance charges and simulate human physiological circumstances, counter ions were added. Throughout the simulation, the NPT ensemble was utilized, with a temperature of 310 K and a pressure of 1 atm. The models were relaxed before the simulation. The stability of the simulation was assessed by contrasting the root mean square deviation (RMSD) of the protein and ligand over time. To create a trajectory of 1000 frames, the structure’s coordinates were saved every 100 ps. Using Maestro’s Simulation Interaction Diagram, post-simulation analyses were carried out [47–49].

## Results

### Computational study of physio-chemicals properties of the SFRP4 Protein

The atomic composition and physio-chemical analyses were done using ProtParam. The atomic composition of SFRP4 showed the presence of 1752 carbon, 2832 hydrogen, 502 nitrogen, 494 oxygen, and 32 sulfur atoms and a formula mass of C1752H2832N502O494S32. The estimated molecular weight of the SFRP4 protein is 39858.75, and 9.12 is its theoretical isoelectric pI. The total integer of negatively charged residues (Asp + Glu) is 37, and the total integer of positively charged residues (Arg + Lys) is 53. The total integer of atoms is 5612, and the atomic composition of all the atoms is given in Table 1. The amino acid composition provides an estimation of the relative abundance of each amino acid within the predicted structure. This information allows us to identify the active site of the protein, which plays a crucial role in its function. The estimated half-life is 30 hours in vitro for mammalian reticulocytes, >20 hours in vivo for yeast, and >10 hours in vivo for Escherichia coli (E, Coli). The aliphatic index of protein is 81.97, and the GRAVY score is -0.421.

**Table 1:** Amino acid composition predicted by ProtParam.

**Ala (A)**	**Asn (N)**	**Cys (C)**	**Glu (E)**	**His (H)**	**Leu (L)**	**Met(M)**	**Pro (P)**	**Thr (T)**	**Tyr (Y)**	**Pyl (O)**
19	12	19	20	10	30	13	30	16	8	0
5.5%	3.5%	5.5%	5.8%	2.9%	8.7%	3.8%	8.7%	4.6%	2.3%	0.0%
**Arg (R)**	**Asp (D)**	**Gln (Q)**	**Gly (G)**	**Ile (I)**	**Lys (K)**	**Phe (F)**	**Ser (S)**	**Trp(W)**	**Val (V)**	**Sec (U)**
25	17	14	6	20	28	5	23	7	24	0
7.2%	4.9%	4.0%	1.7%	5.8%	8.1%	1.4%	6.6%	2.0%	6.9%	0.0%

### Tertiary structure prediction and evaluation

**Fig 2:**
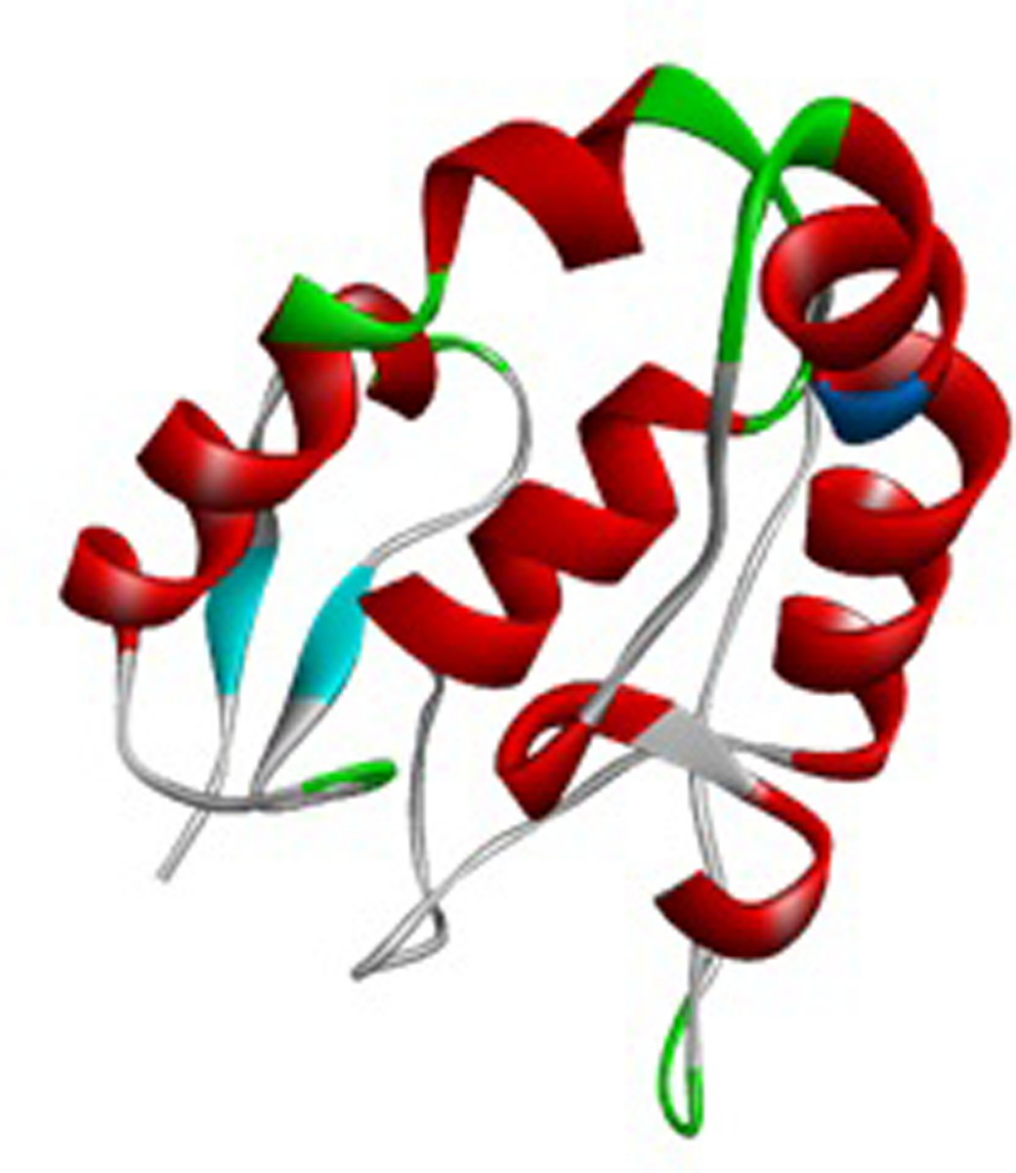
The 3D predicted structure of SFRP4.

**Fig 3:**
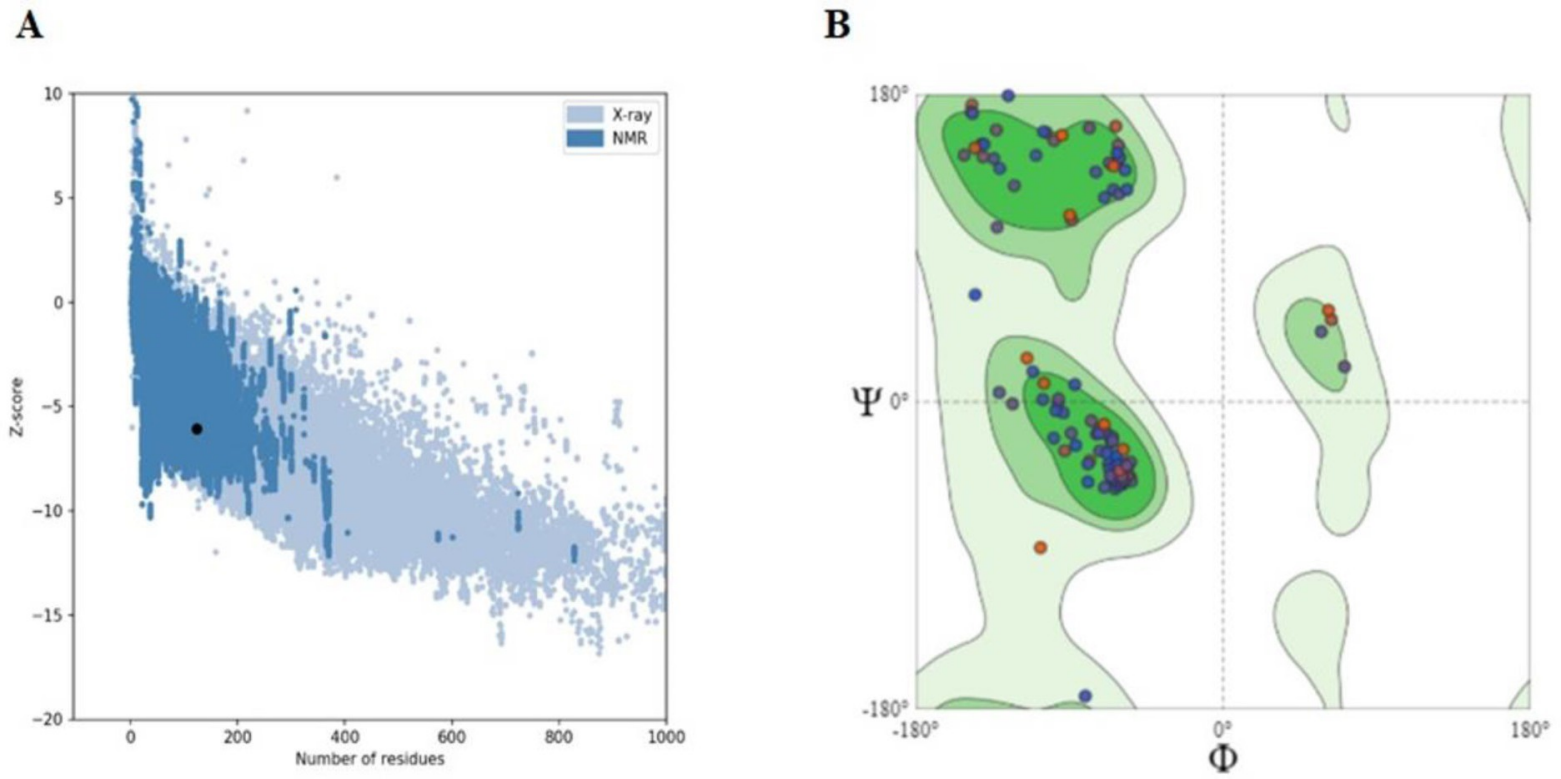
Evaluation of predicted structure. (A) Z-score plot predicated by the proSA website displaying model quality in the X-ray region indicating a high-quality model. (B) The Ramachandran Plot of the SFRP4 structure shows 97.2% residues in the most favored, and 2.8% in the allowed region.

The crystal structure of the SFRP4 protein (Accession no. CAG46532.1) with 346 amino acids was predicted by using the template of secreted frizzled-related protein 3 (1ijx). The selected template showed 73.98% identity with the SFRP4 protein sequence. The QMEAN score of our predicted structure is 0.85 (Fig 2 and S1 Fig in S1 File). The predicted structure was refined by using the online Galaxy Refine web server. The predicted structure Z-score was -6.06. This Z-score value indicated the good quality of the predicted structure by matching the NMR and X-ray structures (Fig 3A and S2 Fig in S1 File). Ramachandran phi/psi plot values indicated good accuracy between the coordinates of the targeted protein. It showed that 97.2% of residues of amino acids were found in the most favored regions, and 2.8% of residues were found in additional allowed regions (Fig 3B and S2 Fig in S1 File). ERRAT showed an overall quality score for the modeled structure with a value of 94.36. The higher the ERRAT quality score, the more significant the quality of the modeled structure. The server VERIFY 3D predicted that in the predicted model, 82.11% of the residues had an averaged 3D–1D score > = 0.2 (Table 2).

**Table 2:** Statistical values of different model evaluation servers.

Predicted Model	Statistics of Ramachandran Plot			ProSA	ERRAT	VERIFY-3D
	Favored	Allowed	Disallowed	z-score	Quality Factor	Compatibility Score (%)
**SFRP4 Protein**	97.2%	2.8%	0.0%	-6.06	94.36	82.11%

### Active site analysis

The active site determined by the online server DoGSiteScorer based on calculated shape, size, and chemical content was confirmed through literature mining. The active pocket residues include Glu60, Tyr61, Glu63, Leu64, Val67, Leu111, Met112, Tyr115, His117, Ser 118, Try119, and Pro 120 [20, 50, 51].

### Protein-ligand interaction analysis

**Fig 4:**
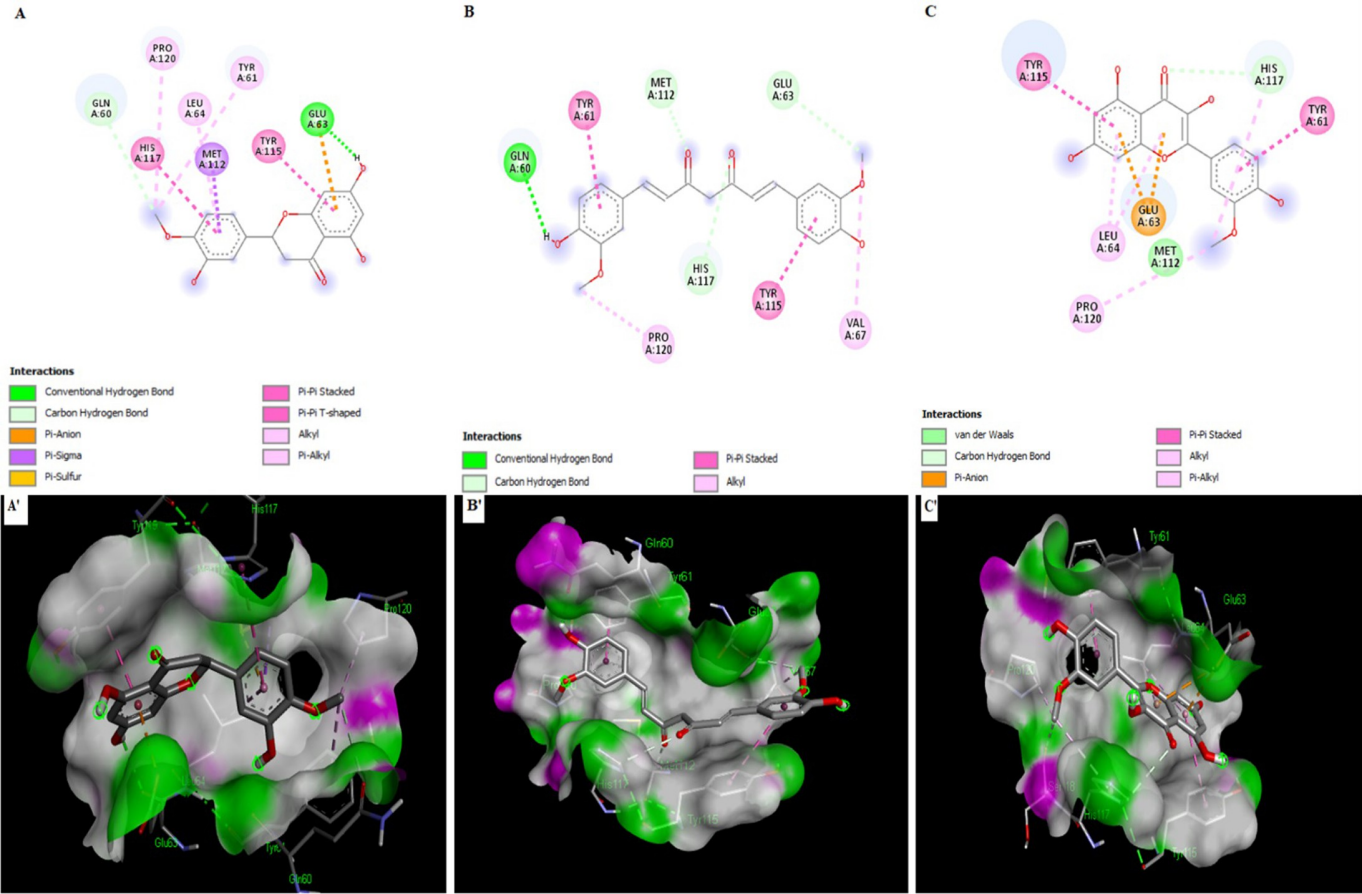
Docking results of various compounds within the active pocket of secreted frizzled-related protein 4 showing ligand interactions (A-C) and Binding Patterns (A’-C’). **(A, A’)** Hesperetin interacted with Gln60, Tyr61, Glu63, Leu64, Met112, Tyr115, His117 and Pro120. **(B, B’)** Curcumin interacted with Gln60, Tyr61, Glu63, Val67, Met112, Tyr115, His117 and Pro120**. (C, C’)** Isorhamnetin interacted with Tyr61, Glu63, Leu64, Met112, Tyr115, His117 and Pro120.

**Fig 5:**
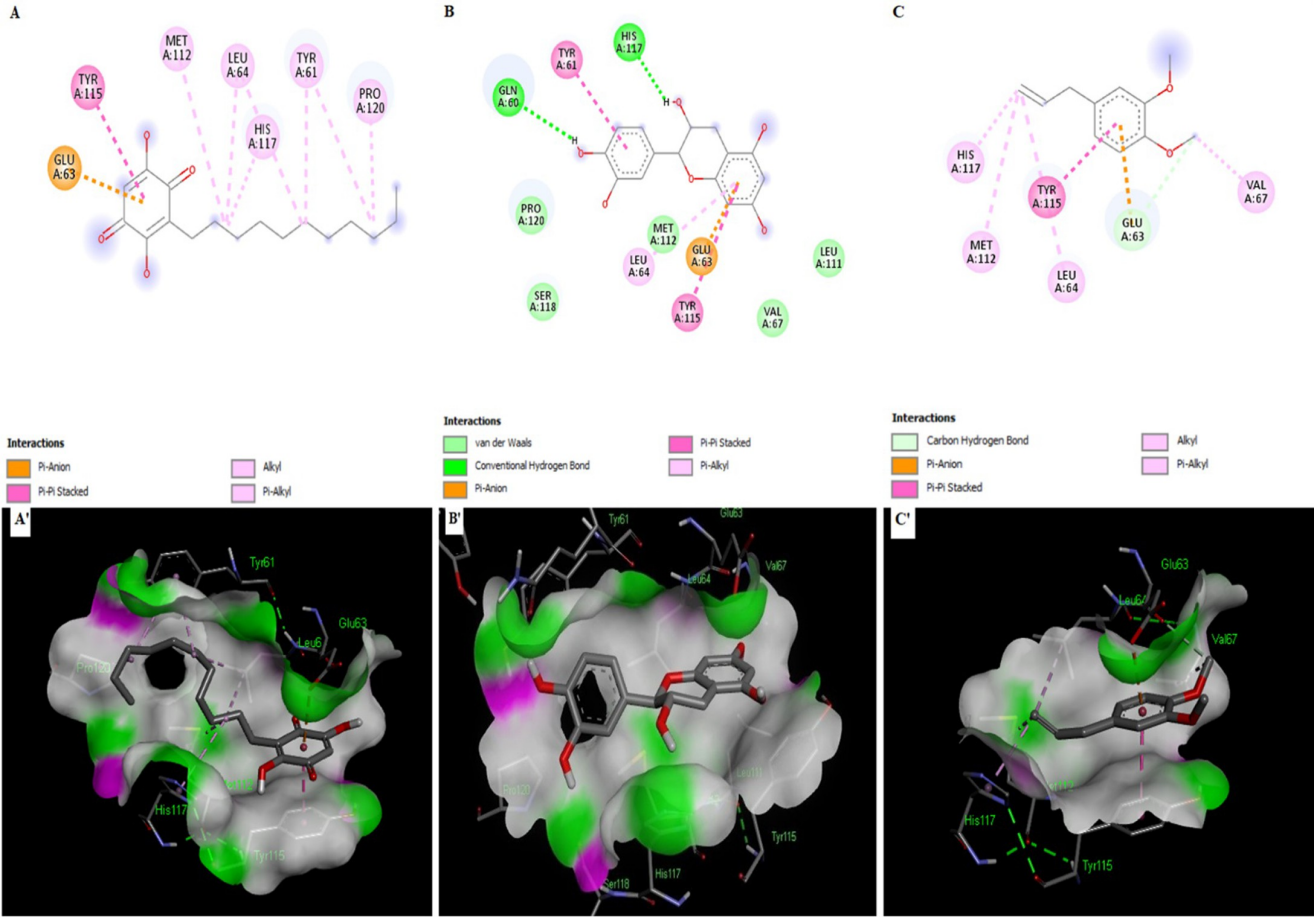
Docking results of various compounds within the active pocket of secreted frizzled-related protein 4 showing ligand interactions (A-C) and Binding Patterns (A’-C’). **(A, A’)** Embelin interacted with Tyr61, Glu63, Leu64, Met112, Tyr115, His117, and Pro120. **(B, B’)** Epicatechin interacted with Gln60, Tyr61, Glu63, Leu64, Tyr115, and His117 while Val67, Leu111, Met112, Ser118, and Pro120 are found in the environmental region. **(C, C’)** Methyl eugenol interacted with amino acids Glu63, Leu64, Val67, Met112, Tyr115, and His117.

After molecular docking of 300+ phytochemicals, the top six phytochemicals were chosen based on their best binding energies, lower RMSD values, their capability to bind well in the pose contrast investigation, and their interaction with active site residues. The lead compound binding energies are shown in Table 3, and the interaction poses are shown in Figs 4 and 5. Hesperetin ligand formed binding interactions with the SFRP4 target protein. These interactions included one conventional hydrogen bond and one carbon-hydrogen bond at Glu63 and Gln60, respectively. Additionally, an electrostatic interaction, Pi-anion, and Pi sigma interaction occurred at Glu63, Met112. Furthermore, Hesperetin was involved in five hydrophobic interactions with Pi-Pi-stacked, Pi-Pi T-shaped, alkyl, and Pi-Alkyl at Tyr115, His117, Pro120, Tyr61, and Leu64 on the active pocket of the receptor protein (Fig 4A and 4A’, S3 Fig in S1 File). The ligand Curcumin with receptor protein revealed one conventional hydrogen at Gln60 and three carbon-hydrogen bond interactions at Glu63, Met112, and His117. There were also four electrostatic interactions with Pi-Pi stacked and Alkyl at Tyr61, Tyr115, Val67, and Pro120 (Fig 4B and 4B’, S4 Fig in S1 File). In the receptor-ligand complex, isorhamnetin formed a carbon-hydrogen bond between isorhamnetin’s oxygen and the residue His117 on the receptor. Additionally, an electrostatic Pi-Anion interaction occurred with Glu63 on the receptor. Five important hydrophobic interactions were observed in the complex, including Pi-Pi-stacked, alkyl, and Pi-alkyl interactions at Tyr115, Tyr61, Pro120, His117, and Leu64. Moreover, Met112 formed a van der Waals bond interaction with the ligand (Fig 4C and 4C’, S5 Fig in S1 File). The ligand Embelin formed a single electrostatic Pi-Anion interaction at Glu63 on the receptor. Moreover, there were six Pi-Pi stacked, Alkyl, and Pi-Alkyl interactions at Tyr115, Pr120, Met112, Leu64, His117, and Tyr61 on the receptor (Fig 5A and 5A’, S6 Fig in S1 File). The ligand epicatechin formed two conventional hydrogen bonds at Gln60 and His117 on the target protein. One electrostatic Pi-Anion interaction is formed at Glu63 and three hydrophobic Pi-Pi-stacked and Pi-alkyl interactions are formed at Tyr61, Tyr115, and Leu64 amino acids on the target protein. Additionally, there were four amino acids involved in van der Waals, including Val67, L111, Met112, Ser118, and Pro 120 (Fig 5B and 5B’, S7 Fig in S1 File). The ligand methyl eugenol formed one-carbon hydrogen and Pi-Anion bond at Glu63. There were five Pi-Pi Stacked, alkyl, and Pi-alkyl bonds formed with Tyr115, Val67, Met112, Leu64, and His117 on the receptor protein (Fig 5C and 5C’, S8 Fig in S1 File).

**Table 3:** Details and the structures of the top six phytochemical docking interactions with bond distance in the SFRP4 protein’s active pocket.

Sr. No.	Ligand Name	Molecular Formula	Binding Score (Kcal/mol)	Interacting Residues in the Active Pocket	Residues Bond Interaction Distance (Å)
**01**	Hesperetin	C_16_H_14_O_6_	-6.3	Gln60	2.54
				Tyr61	4.87
				Glu63	3.36
				Leu64	4.85
				Met112	3.83;4.19
				Tyr115	3.93
				His117	4.95
				Pro120	4.77
**02**	Curcumin	C_21_H_20_O_6_	-6.4	Gln60	2.07
				Tyr61	4.97
				Glu63	3.73
				Val67	4.82
				Met112	3.61
				Tyr115	4.12
				His117	3.63
				Pro120	4.22
**03**	Isorhamnetin	C_16_H_12_O_7_	-6.3	Tyr61	5.10
				Glu63	3.27;4.00
				Leu64	5.25;5.42
				Met112	0
				Tyr115	4.57
				His117	4.69;3.39
				Pro120	4.70
**04**	Embelin	C_17_H_26_O_4_	-5.3	Tyr61	3.29
				Glu63	3.29
				Leu64	5.01;4.65
				Met112	4.89
				Tyr115	3.69
				His117	4.45
				Pro120	4.35
**05**	Epicatechin	C_15_H_14_O_6_	-6.3	Gln60	2.91
				Tyr61	5.26
				Glu63	3.29
				Leu64	5.21
				Tyr115	4.60
				His117	2.17
**06**	Methyl Eugenol	C_11_H_14_O_2_	-5.8	Glu63	3.44
				Leu64	5.05
				Val67	5.15
				Met112	4.49
				Tyr115	3.89
				His117	3.80

### Drug-likeness and ADME/T profiling

The top six ligands based on good binding affinities against SFRP4 showed zero violation of the Lipinski rule as given in Table 4. Furthermore, all six ligands were submitted to different servers for ADMET profiling to validate the drug-like behavior. The ADMET profiling results of different servers are summarized in Table 5. All of the selected ligands demonstrated very good intestinal solubility and positive CaCO_2_ permeability and in terms of distribution, not all of them are permeable to cross the blood-brain barrier. The ligands were also auspicious towards cytochrome enzymes P450 having shown few possible inhibitions to *CYP1A2*, *and CYP2C19*. Renal and total clearance were also low for all the lead ligands. Finally, in terms of toxicity prediction results of selected lead ligands passed the AMES test for toxicity. However, the overall results of these lead ligand ADMET drug screenings were acceptable, and these leads might be considered effective therapeutic candidates against SFRP4.

**Table 4:** Molecular properties of top six ligands.

Phytochemical Name	Molecular Properties						
	Molecular Weight (<500 Dalton)	HBD	HBA	nrotb	Log P	A	Violations
**Hesperetin**	302.2	3	6	2	2.473	78.06	0
**Curcumin**	368.4	2	6	8	2.742	102.80	0
**Isorhamnetin**	316.2	4	7	2	2.541	82.50	0
**Embelin**	294.4	2	4	10	4.935	84.31	0
**Epicatechin**	290.2	5	6	1	1.142	74.33	0
**Methyl Eugenol**	178.2	0	2	4	2.540	53.53	0

**HBD:** Number of hydrogen bond donors, **HBA:** Number of hydrogen bond acceptors, **nrotb:** Number of rotatable bonds, **A:** molar refractivity, **log P:** The logarithm of octanol/water partition coefficient.

**Table 5:** ADMET analysis of the top six ligands.

Properties		Hesperetin	Curcumin	Isorhamnetin	Embelin	Epicatechin	Methyl Eugenol
**Absorption**	*Water Solubility*	-3.478	-4.01	-3	-2.395	-3.101	-2.737
	*Caco2 permeability (Log Papp in 10*^*−6*^ *cm/s)*	0.466	-0.093	-0.003	0.956	0.38	1.503
	*Intestinal absorption (% absorbed)*	90.28	82.19	76.014	97.32	89.32	99.66
	*Skin Permeability (Log Kp)*	-3.672	-2.735	-2.735	-2.949	-3.603	-1.795
	*P-glycoprotein substrate*	Yes	Yes	Yes	Yes	Yes	Yes
**Distribution**	*BBB permeability (Log BB)*	-1.013	-0.215	-1.135	-0.332	-0.905	0.578
	*CNS permeability (Log PS)*	-2.964	-2.99	-3.188	-2.675	-3.146	-1.795
**Metabolism**	*CYP 2D6 Substrate*	No	No	No	No	No	No
	*CYP 3A4 Substrate*	No	Yes	No	No	No	No
	*CYP 1A2 Inhibitor*	No	Yes	Yes	No	No	Yes
	*CYP 2C19 Inhibitor*	No	Yes	No	Yes	No	No
	*CYP 2C9 Inhibitor*	No	Yes	No	No	No	No
	*CYP 2D6 Inhibitor*	No	Yes	No	No	No	No
	*CYP 3A4 Inhibitor*	No	Yes	No	No	No	No
**Excretion**	*Total Clearance (Log ml/min/kg)*	0.044	-0.002	0.508	1.313	0.215	0.325
	*Renal OCT2 substrate*	No	No	No	No	No	No
**Toxicity**	*AMES toxicity*	No	No	No	No	No	No
	*Skin Sensitization*	No	No	No	Yes	No	Yes
	*Oral Rat Acute Toxicity (LD50) (mol/kg)*	2.221	1.833	2.407	2.087	2.221	2.005

### MD simulation analysis

**Fig 6:**
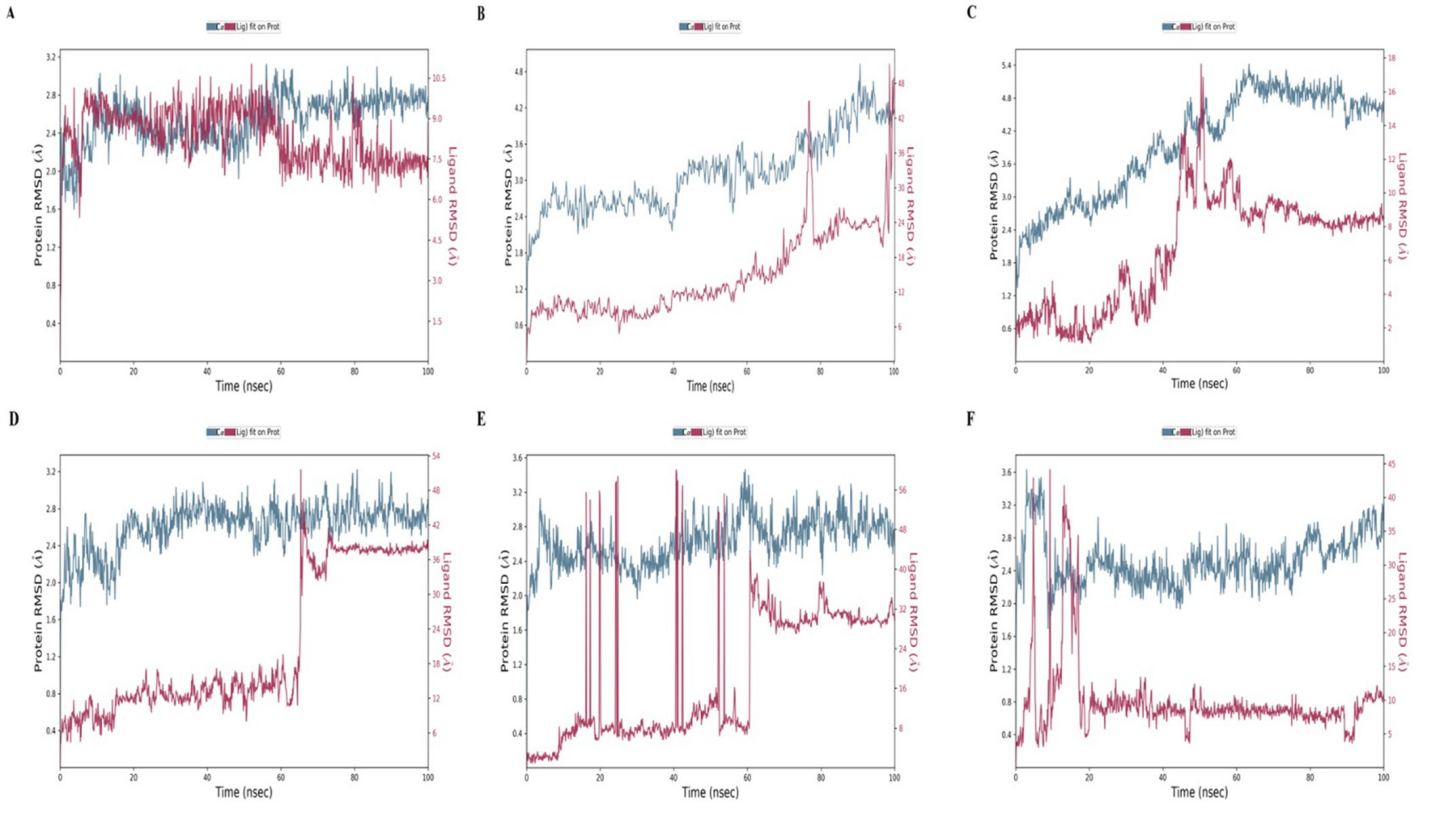
MD Simulation RMSD Plot of C-alpha atoms in proteins and ligands **(A)** Hesperetin **(B)** Curcumin **(C)** Isorhamnetin **(D)** Embelin **(E)** Epicatechin **(F)** Methyl eugenol. The left-hand frames show the Protein RMSD (P-RMSD) value over time, whereas the right-hand frame shows the ligand RMSD (L-RMSD) value over time.

**Fig 7:**
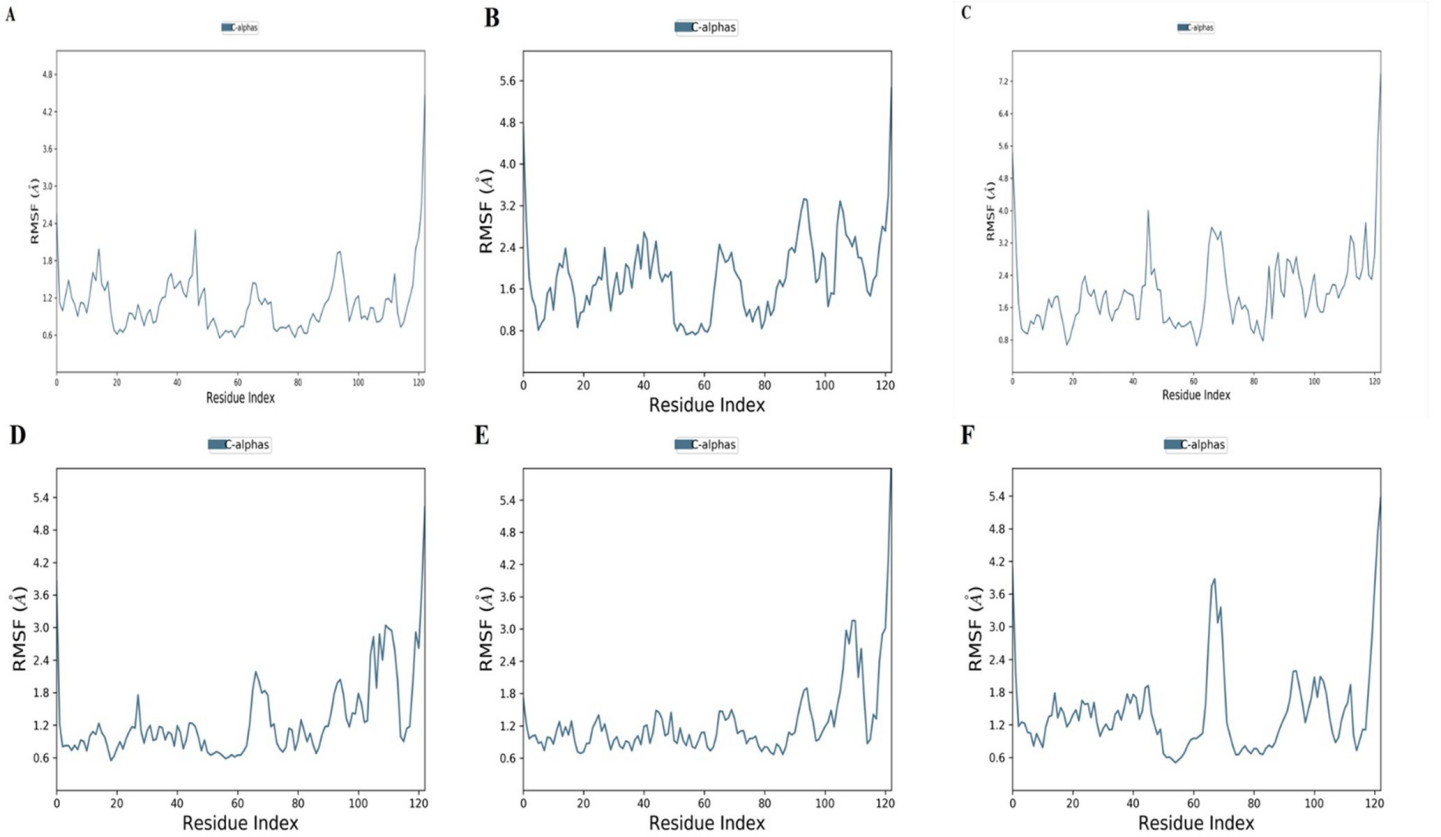
MD Simulation P-RMSF Plot of complexes (A) Hesperetin (B) Curcumin (C) Isorhamnetin (D) Embelin (E) Epicatechin (F) Methyl eugenol.

**Fig 8:**
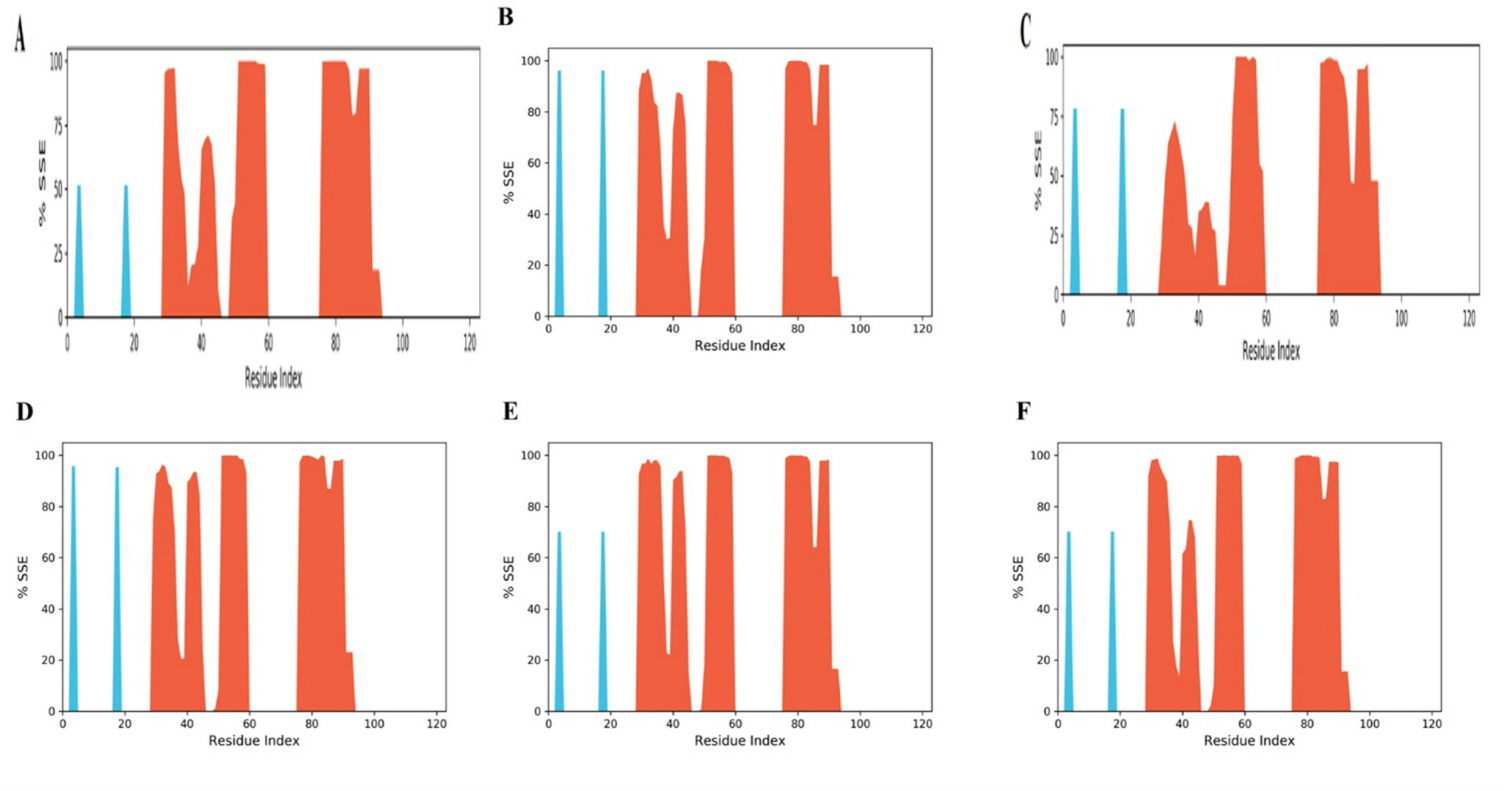
The distribution of protein secondary structure elements in protein structures complexed with ligands by residue index. The red columns represent alpha helices, whereas the blue columns represent beta strands **(A)** Hesperetin **(B)** Curcumin **(C)** Isorhamnetin **(D)** Embelin **(E)** Epicatechin **(F)** Methyl eugenol.

All the docking results showed a good binding affinity with SFRP4 then to check the stability of binding MD simulation was performed by Desmond at 100ns. The conformational changes, stability, and major binding events of the selected ligands within the receptor’s binding pocket were assessed. After performing the simulation analysis results of the simulation trajectories were used for several dynamics analyses, including root mean square fluctuation (RMSF), root mean square deviation (RMSD), protein secondary structure elements, and Protein-ligand interactions (Figs 6–8). The comparative analysis demonstrated that, in comparison to other complexes, the Hesperetin complex was more stable during the course of the simulation, which justified stability and supported our docking findings.

### Root Mean Square Deviation (RMSD) analysis

Protein RMSD of the Hesperetin-SFRP4 complex attained stability at 10 ns and maintained it until the end of the 100 ns MD simulation. P-RMSD of the Hesperetin system lies in the range of ∼2.8–3.0 Å. Similarly, the ligand RMSD of the Hesperetin system stabilizes from 10 ns to 58 ns. After 58 ns, L-RMSD slightly decreases but maintains stability. At 80 ns, hesperetin experiences a peak due to five rotatable bonds and again stabilizes over the simulation (Fig 6A and S9 Fig in S1 File). The P-RMSD of the curcumin complex in blue remains stable till 40 ns and then gradually increases till 4.2 Å over simulation with no major deviation, while the ligand in contact with the receptor remains stable till 77 ns, then experiences a larger peak and again attains stability and maintains it till 98 ns (Fig 6B and S10 Fig in S1 File). The P-RMSD value in the isorhamnetin complex starts increasing slowly from the start of the simulation at 2.4 Å. It stabilizes at 65 Å and fluctuates within a range of 1 Å. Then, at 65 ns, RMSD started dropping slowly, and at the end, its value was 4.8 from 5.4 Å. This complex’s ligand-fit protein RMSD values varied within 1.5 Å until 35 ns, then increased by 10 Å (this could be due to the ligand mode flip). The RMSD then achieved equilibrium at 60 ns and stayed constant for the remainder of the MD experiment (Fig 6C and S11 Fig in S1 File). The ligand Embelin P-RMSD at the start exhibited a little fluctuation, then stabilized at 20 ns and maintained as such till the end of the simulation within the acceptable range of 3.2 angstroms. Conversely, within the L-RMSD embelin complex system, consistent stability is observed up to 64 ns. After that, it experiences a larger peak at 64 ns, and after 65 ns, it slightly decreases. Again, it attains stability at 72 ns and maintains this stability till the end of the 100 ns simulation (Fig 6D and S12 Fig in S1 File). In the Epicatechin complex system, P-RMSD remains stable until 56 ns, and at 58 ns, it experiences a slight peak within an acceptable range of 3.6 Å, and after that, it maintains stability till the end of the simulation. L-RMSD of the epicatechin complex experiences a higher peak due to six rotatable bonds. At 60 ns, it gets stable throughout the MD simulation (Fig 6E and S13 Fig in S1 File). In the case of the methyl eugenol complex, P-RMSD stabilizes at 10 ns and maintains till the end of the simulation, and the same happens in the case of the ligand-fit protein, which attains stability at 20 ns and maintains it over a 100 ns simulation (Fig 6F and S14 Fig in S1 File).

### Root Mean Square Fluctuation (RMSF) analysis

The RMSF (root mean square fluctuation) of the receptor in contact with ligands was calculated to assess the mobility of each residue. There are some higher peaks, and the residues of the higher peaks represent loops, coils, and turns. These peaks represent more flexibility than other regions of the protein. In this respect, it is worth noting that the residues with greater peaks are generally found in loop regions of the protein’s N and C termini. As well, the N and C terminal regions depict more variations. These results are based on an examination of MD trajectories. The P-RMSF of the hesperetin complex fluctuates within ∼2.4 Å, with four smaller peaks that lie in an acceptable range and one higher peak at the C terminal (Fig 7A and S15 Fig in S1 File). The curcumin SFRP4 complex P-RMSF shows continuous peaks with more fluctuations than hesperetin, with a 3.5 Å RMSF value (Fig 7B and S16 Fig in S1 File). The complex isorhamnetin P-RMSF shows continuous peaks and fluctuations. It also exhibited two higher peaks at residues 43 and 62 in the range of 4.0 Å and 3.5 Å (Fig 7C and S17 Fig in S1 File). The P-RMSF of embelin exhibits consistent peaks from the start to residue 60 in the range of 1.8 Å, then it shows two notable fluctuations at residues 65 as well as at residues 110 to 118 with values around 3.0 Å (Fig 7D and S18 Fig in S1 File). The epicatechin complex shows stability with two higher peaks at the N termini (Fig 7E and S19 Fig in S1 File). Lastly, the P-RMSF of the methyl eugenol complex lies within ∼1.8 Å and then experiences a larger fluctuation. Again, this system gains stability until residue 115 (Fig 7F and S20 Fig in S1 File). Resultantly, the hesperetin system exhibits fewer motions than others over a 100 ns MD simulation, which suggests more secure and tighter ligand-receptor interactions. All other systems show variation within an acceptable range, except for the loop regions.

### Secondary Structure Elements (SSEs) analysis

Secondary structure elements (SSE) exhibit structural stability, the distribution of different structural elements over time, and ligand-receptor interactions during simulation. In the case of hesperetin, it was found that 28.07% of the secondary structure of this complex was made up of helixes. This shows that some parts of the protein have an alpha-helical shape. Strands, on the other hand, accounted for 1.68% of the total, reflecting areas with a beta-strand or beta-sheet conformation. The total composition consisting of secondary structural components within the hesperetin complex was 29.75% (Fig 8A and S27 Fig in S1 File). In the curcumin SFRP4 complex, there were 32.82% of total SSEs (Fig 8B and S28 Fig in S1 File). In the Isorhamnetin complex, it was found that there are a total of 27.91% SSEs (Fig 8C and S29 Fig in S1 File). In complex embelin (32.98%), epicatechin (32.38%), and methyl eugenol (31.33%), secondary structure elements were found (Fig 8D-8F S30-S32 Figs in S1 File). Different SSE percentages for each complex are due to interaction with different ligands in distinct ways. Information on the stability of complexes was gained via secondary structure element analysis, as they didn’t make much noise compared to loops, coils, and turns. Resultantly. The major portion of the receptor in all systems consists of alpha helices and a smaller proportion of beta strands (S27-S32). The information gained via SSE analysis is employed to predict new drug molecules that alter protein function in a desired way.

### Protein-Ligand contacts analysis

**Fig 9:**
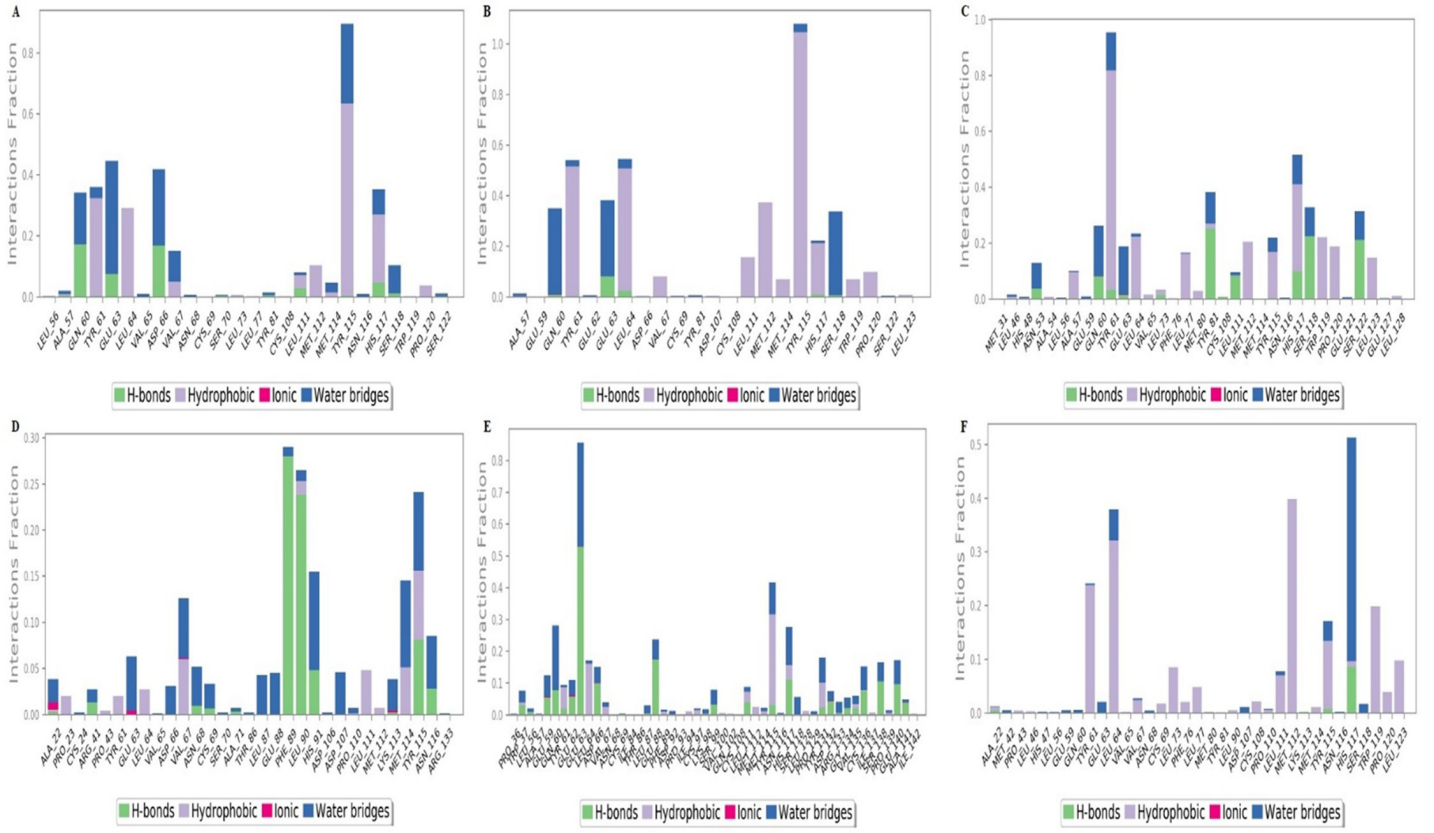
The Protein-Ligand contacts of lead complexes **(A)** Hesperetin **(B)** Curcumin **(C)** Isorhamnetin **(D)** Embelin **(E)** Epicatechin **(F)** Methyl eugenol.

By using the MD simulation, various intermolecular interactions, including hydrogen bonds, water bridges, hydrophobic interactions, and ionic interactions, were thoroughly examined to critically evaluate their significance. Protein-ligand interactions are summarized in the form of a plot. Interaction types are categorized with different colors during MD simulation. The noteworthy hydrogen bonds (Gln60 and Glu63) were identified in the initial docked configuration of the hesperetin compound they remained unchanged during the simulation period. Hydrophobic interactions were noticed with residues Tyr61, Leu64, Tyr115, and His117. Water bridges were most significantly observed with the residues Gln60, Glu63, Asp66, and Tyr 115 (Fig 9A and S21 Fig in S1 File). The curcumin complex with SFRP4 formed hydrogen bonds with the residue Glu63. The residues Gln60, Leu64, Met112, Tyr115, and His117 showed hydrophobic interactions. The residues Gln60, Glu63, and Ser118 formed water bridges (Fig 9B and S22 Fig in S1 File). The isorhamnetin SFRP4 complex showed hydrogen bonding with residues Tyr81, Ser118, and Ser122. The residues Tyr61, Leu64, His117, Trp119, and Pro120 showed hydrophobic interactions. This complex also showed water bridges with residues Gln60 and Glu63 (Fig 9C and S23 Fig in S1 File). The embelin complex demonstrated significant hydrogen bonding with residues Phe89, Leu90, and Tyr115. The residues Val67, Met114, and Tyr115 showed hydrophobic interactions. This complex showed water bridges with residues Glu63, Val67, His91, and Met114. It also exhibited ionic interaction with residues Ala22, Glu63, and Lys113 (Fig 9D and S24 Fig in S1 File). The epicatechin-SFRP4 complex showed hydrogen bonding interactions with residues Glu63, Glu88, His117, and Glu140. It showed hydrophobic interactions with residues Leu64 and Tyr115. The residues Gln60 and Glu63 also formed water bridges (Fig 9E and S25 Fig in S1 File). The methyl eugenol demonstrated hydrophobic interactions with the residues Tyr61, Glu63, Met112, Tyr115, and Trp119. It also showed significant water bridge interactions with the residue His117 (Fig 9F and S26 Fig in S1 File).

## Discussion

Diabesity refers to the simultaneous coexistence of challenging diseases like obesity and diabetes within one individual. Over the world, the prevalence of T2DM and obesity is rising quickly [3]. T2DM is characterized by an increase in blood sugar levels caused by insufficient pancreatic insulin production which as a result leads to hyperglycemia [52, 53]. Insulin resistance, diabetes mellitus, and obesity are closely related to metabolic issues [13]. T2DM, fatty liver disease, hypertension, steatohepatitis, and dyslipidemia are serious health consequences that can arise from obesity [54]. It affects people of all ages, but more frequently women and children than men [55].

It was found that T2DM patients had increased blood concentrations of SFRP-4 even before the development of hyperglycemia [19]. SFRP-4 disturbs the emission of insulin in humans as an extra-cellular controller of the Wingless pathway [56]. SFRP-4 contains a cysteine-rich domain of about 110–120 amino acids and it was 30–40% similar to the Wnt ligand binding domain of the frizzled [57].

Natural products are gaining more and more attention for their potential applications in the prevention and treatment of type 2 diabetes mellitus and obesity. These products include plant extracts, phytochemicals, and microbial metabolites. The development of drugs from natural sources is a significant and quick-creating zone. Thousands of year’s plants have been used to cure a range of diseases including diabetes and obesity [58]. Several plant extracts and their phytochemicals have been tested for their anti-diabesity properties using *in silico* approaches. The phytochemicals identified in the current study demonstrated very promising effects, indicating that dietary photochemical intake could be a promising strategy for obesity and diabetes prevention [59].

Our docking results of six reported leads showed the strongest interaction against SFRP4 protein with the closest RMSD values and the most active site residues of amino acids participating in the interaction. Lead one Hesperetin was found to be the utmost potential inhibitor based on the number of interactions with amino acids in an active pocket. It is a trihydroxy flavanone with three hydroxy groups, and it possesses antioxidant, anti-allergic, anti-inflammatory, Vaso-protective, and hypolipidemic characteristics. The natural sources of Hesperetin are citrus fruits, which are also utilized to decrease cholesterol levels [60]. Flavanone-rich supplements like hesperetin are the most important dietary phytomedicine. In both in vivo and in vitro models, it protects pancreatic beta cells from death by modulating AMPK (AMP-activated protein kinase)-mediated p300 inactivation under severe hyperglycemic conditions and in the late stages of diabetes. [61, 62]. Hesperetin is effective in mitigating hyperglycemia by potentially promoting the release of insulin from the beta cells within the islets. In streptozotocin-induced rats, treatment with hesperetin was found to be effective in preserving the usual histological manifestations of renal, hepatic, and insulin-positive beta-cells [63]. Hesperetin supplements effectively decrease carbohydrates, lipid metabolism, oxidative stress, inflammation, IR, and PI3K expression levels. It has beneficial antidiabetic effects on type 2 diabetic rats by affecting the AMPK-mediated signaling pathway in the pancreas by inhibiting the production of TNF-α, IL-6, caspase-3, IL-1, and apoptosis, and restoring pancreatic beta cells [64]. All the metabolites of flavanone improved insulin secretion, mitochondrial function, and reduced apoptosis. Therefore, hesperetin could reduce oxidative stress in β-cells and improve their function [65]. Lead two curcumin phytochemical is found in the Curcuma longa with anti-inflammatory, antioxidant, antimicrobial, and anticancer properties that may be useful in treating diabetes complications. Curcumin has been shown to reduce the levels of glycated hemoglobin, total cholesterol, fasting blood glucose, serum C reactive protein, triglycerides, and body mass index. As a result, curcumin has the potential to be used as a therapeutic agent for diabetic patients [66]. Lead three Isorhamnetin is the metabolite of quercetin found extensively in apples, blackberries, and the herbal plant Hippophaer hamnoides L.) [67]. Isorhamnetin has a wide range of pharmacological properties, including anti-inflammatory, antioxidant, anticancer, anti-diabetic, and anti-obesity [68]. Lead four Embelin is studied in many types of cancer treatment, both *in vitro* and *in vivo* experiments. It can efficiently block the proliferation and migration of malignant cells. Embelin has been utilized as an anthelminthic treatment and is successful in treating rats with acetic acid-induced ulcerative colitis. Additionally, it reduced the total cholesterol and triglycerides, heart rate, systolic blood pressure, and systolic blood pressure in diabetic rats [69]. Lead five Epicatechin is a flavonoid that is found in abundant amounts in nature, and diabetic patients, it plays an important role in the decrease of blood glucose levels. Additionally, it has also demonstrated antiangiogenic, anti-oxidant, and antiproliferative properties against cancer cells [70]. Lead six is methyl eugenol, and its therapeutic use results in a reduction in the levels of uronic acid, total cholesterol, total lipid, and abstinence from blood sugar in both normal and alloxanized mice. To interfere with the absorption of glucose, produce and reinvent insulin, and for insulin action, methyl eugenol excites the pancreas [71].

All these leads support its additional role and increased secretion of insulin by modulating and inhibiting the activity of SFRP4, following the results of Yasmin et al., 2020; Hassan et al., 2018; and Bukhari et al., 2014. In the interaction studies, the SFRP4 protein’s His 117, Ser 118, Tyr 61, Ser 122, Ser 118, Gln 60, and Pro 120 amino acid residues were the most reactive, and they participated in interactions more frequently than other amino acid residues, in accordance to results by Yasmin et al., 2020, Hassan et al., 2018 and Bukhari et al., 2014. Then these leads were subjected to MD simulation, a standard method used in molecular modeling, to further validate or examine our results of docking. To determine the stability of the docked protein-ligand complexes, a 100 ns MD simulation was run using the Schrodinger module Desmond, and certain parameters, such as RMSD, were used to examine the stability. The simulation findings demonstrated that the phytochemicals Hesperetin and Epicatechin, respectively, exhibited good stability with SFRP4 during the simulation time, and these results supported their inhibitory potential in accordance with the results of Yasmin et al., 2020; Hassan et al., 2018; Bukhari et al., 2014 [20, 50, 51]. However, the lead Hesperetin appeared more stable during the simulation period, demonstrating that the phytochemical Hesperetin is the most promising option for acting as an SFRP4 inhibitor. Hesperetin might interfere with the activity of SFRP4, which is released from both alpha and beta cells and contributes to beta-cell dysfunction. We can speculate that hesperetin’s anti-inflammatory properties might aid in reducing the inflammation that SFRP4 causes because SFRP4 is associated with low-grade islet inflammation in T2D. This could contribute to the preservation of beta-cell function by reducing the negative impact of chronic inflammation on insulin production and glucose metabolism, leading to better insulin release and glucose control. Plant-based compounds cause proper insulin production, which is dynamic for the treatment of diabesity. To create efficient therapy options for the treatment of diabesity, additional *in vitro* and *in vivo* investigations are required to confirm the antidiabesity properties of these phytochemicals.

## Conclusion

SFRP4 is an inflammatory mediator and a potential target for preserving β-cell function and acts as an early biomarker for the treatment of obesity-induced diabetes (diabesity). In this current study, the CADD approaches are used to identify promising phytochemical candidates for modulating SFRP4—a potential avenue for treating obesity-induced diabetes. The three-dimensional structure of SFRP4 was predicted, and molecular docking was performed to investigate its interactions with phytochemical inhibitors. In molecular docking, six phytochemicals, including hesperetin, curcumin, isorhamnetin, embelin, epicatechin, and methyl eugenol, showed strong interactions with the active site residues Gln60, Glu63, Tyr61, Leu64, Met112, Tyr115, His117, and Pro120 of SFRP4. Based on binding interactions, binding affinities, and ADMET analysis all six docked complexes were utilized in 100ns MD simulations to check the good dynamics and stability within the SFRP4 active site. The top six phytochemicals found in our study have the ability to bind with the SFRP4 protein and might inhibit the activity of the protein. However, predicted RMSD and RMSF graphs justified the finding that hesperetin had a better potential to inhibit the SFRP4 protein as compared to other compounds. These phytochemicals can be used as an antidiabesity compound directly from the plant extracts to control obesity-induced diabetes (diabesity). In short, the comprehensive evaluation of all tested drug-like phytochemicals offers a promising avenue in the quest for discovering a novel treatment against diabesity.

## Supporting information

S1 File (DOCX)
