# Supplementary material for: In Silico identification of novel phytochemicals that target SFRP4: An early biomarker of diabesity
Source: PLoS One. 2023 Nov 9;18(11):e0292155. doi: 10.1371/journal.pone.0292155 (PMC10635506; doi:10.1371/journal.pone.0292155)
Supplement: S1 File — (DOCX) [file pone.0292155.s001.docx]

***In Silico* identification of novel phytochemicals that target SFRP4: An early biomarker of diabesity**

**Asim Rehman^1^, Shazia Anwer Bukhari*^1^, Naheed Akhter^1^, Muhammad Abdullah Ijaz Hussain^2^, Zunera Chauhdary^3^**

1. Department of Biochemistry, Government College University Faisalabad, Faisalabad 38000, Pakistan.
2. Department of Chemistry, Government College University Faisalabad, Faisalabad 38000, Pakistan.
3. Faculty of Pharmaceutical Sciences, Government College University Faisalabad, Faisalabad 38000, Pakistan.

**Corresponding author**

Shazia Anwer Bukhari; Email: shaziabukhari@gcuf.edu.pk

**Supplementary Data**

**
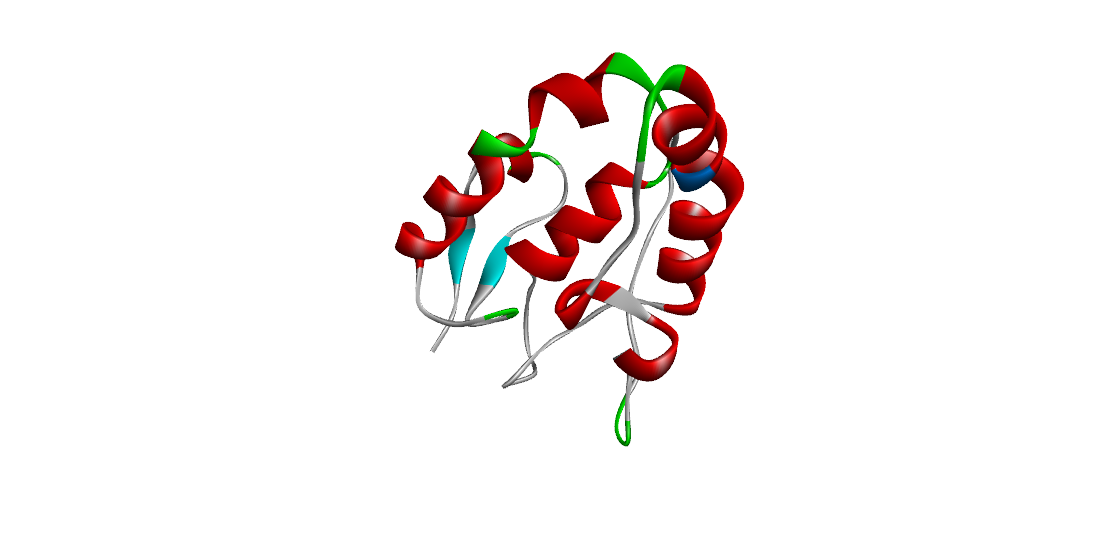
**

**Fig S1:** 3D Predicted structure of SFRP4 Protein.

| **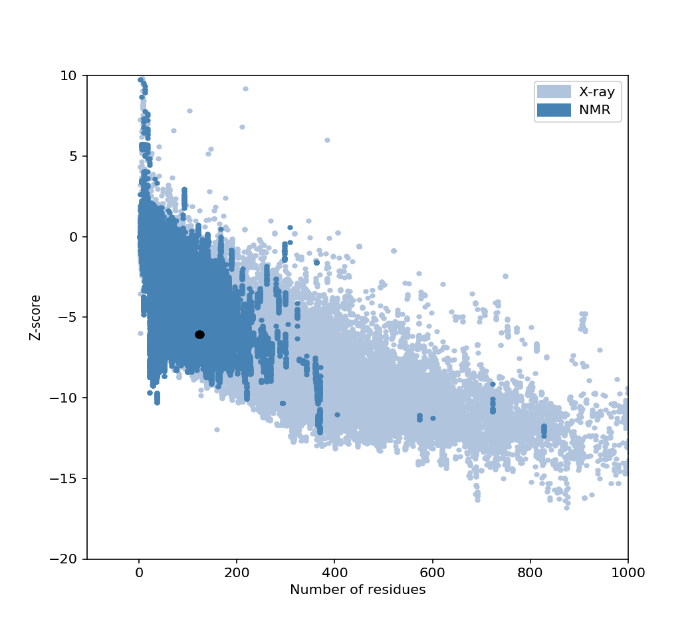**  **(B)**  **(A)** | 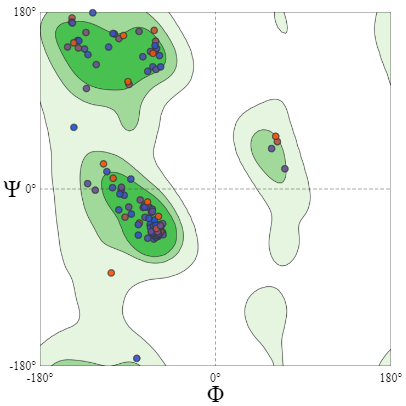 |  |
| --- | --- | --- |

**Fig S2**: Evaluation of predicted structure **(A)** proSA Z-score and **(B)** Ramachandran plot of SFRP4 Protein.

| **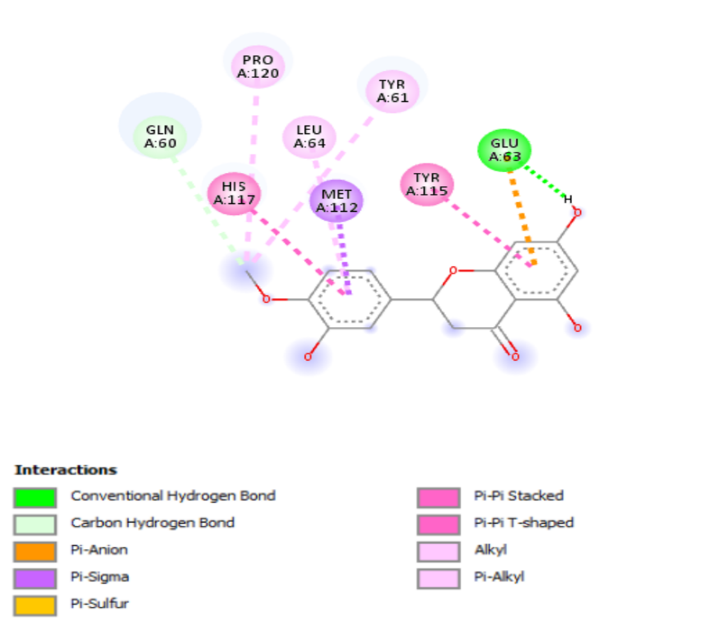**  **(B)**  **(A)** | 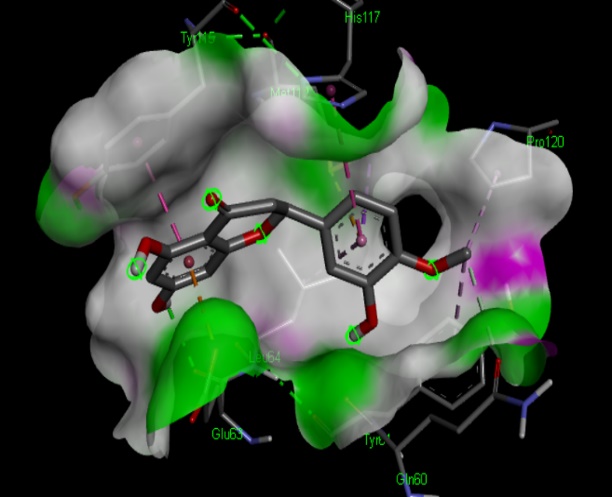 |  |
| --- | --- | --- |

**Fig S3:** Interaction **(A)** and binding pattern **(B)** of Hesperetin with SFRP4 Protein.

| **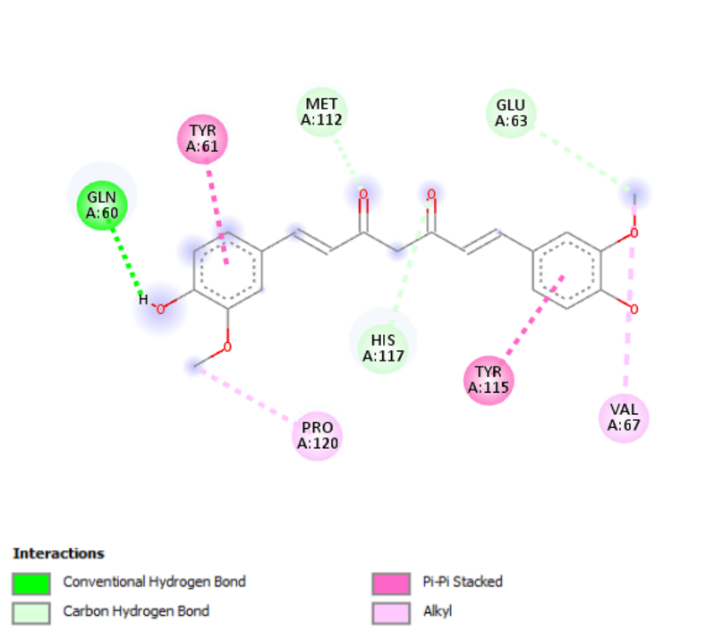**  **(B)**  **(A)** | 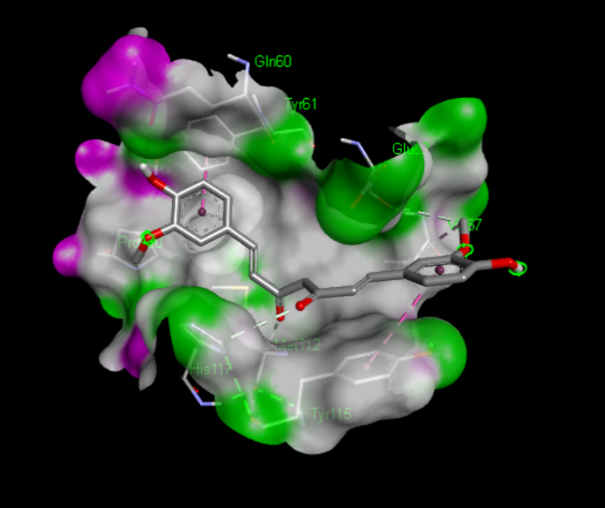 |  |
| --- | --- | --- |

**Fig S4:** Interaction **(A)** and binding pattern **(B)** of Curcumin with SFRP4 Protein.

| **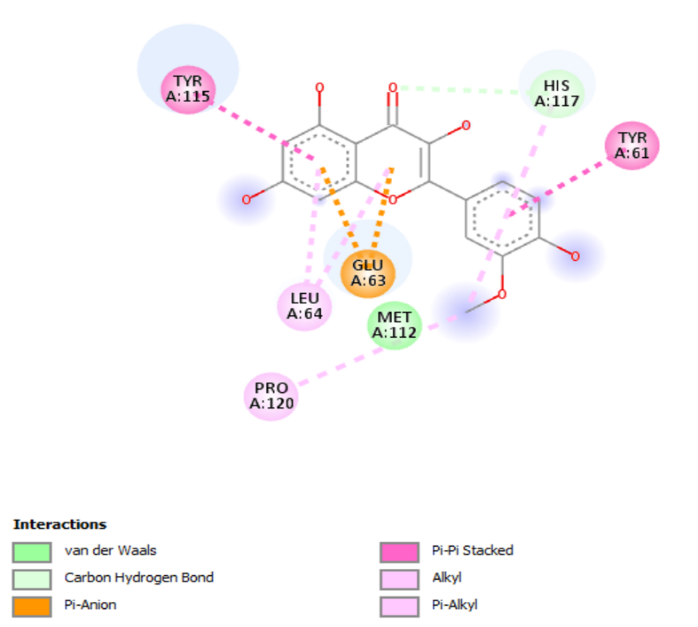**  **(B)**  **(A)** | 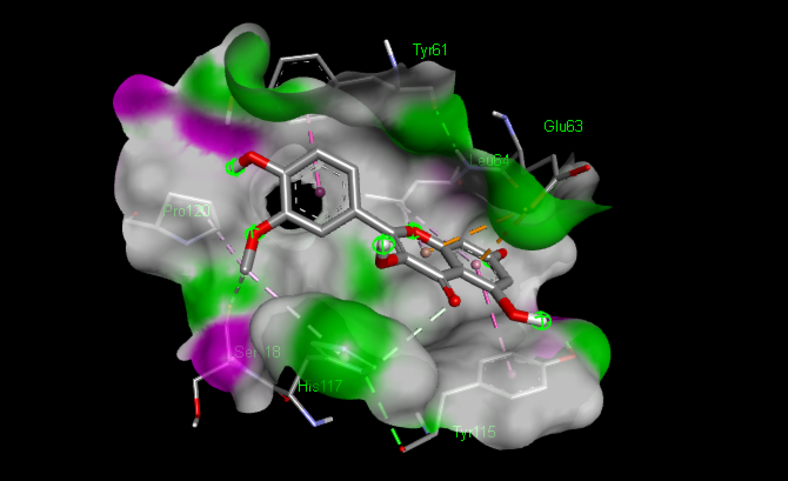 |  |
| --- | --- | --- |

**Fig S5:** Interaction **(A)** and binding pattern **(B)** of Isorhamnetin with SFRP4 Protein.

| **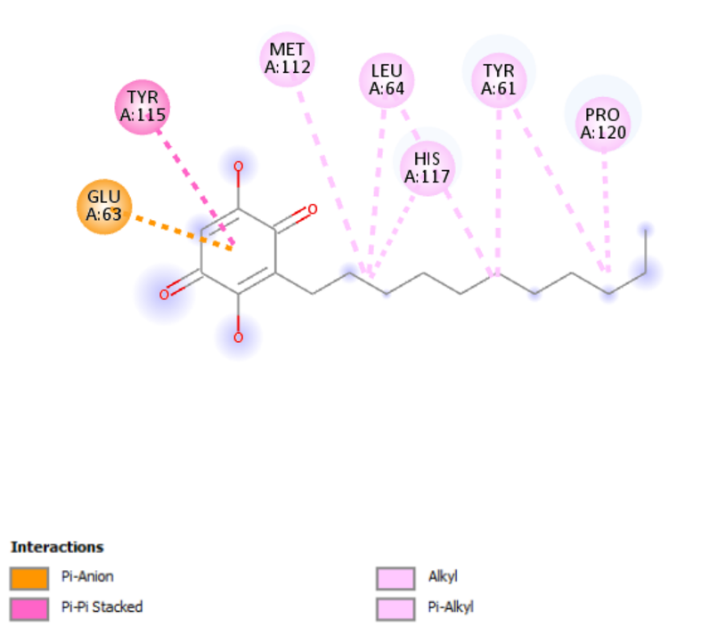**  **(B)**  **(A)** | 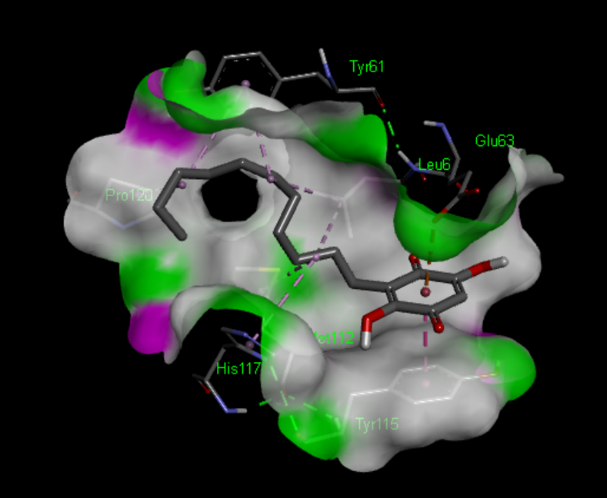 |  |
| --- | --- | --- |

**Fig S6**: Interaction **(A)** and binding pattern **(B)** of Embelin with SFRP4 Protein.

| **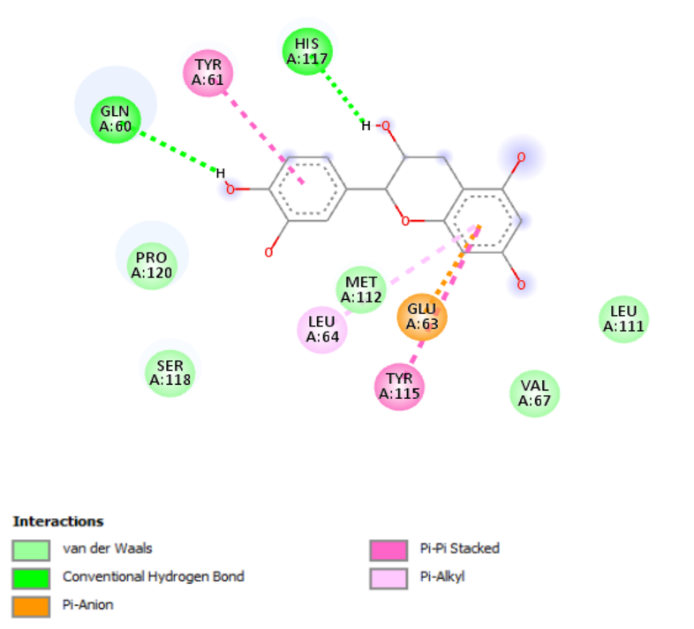**  **(B)**  **(A)** | 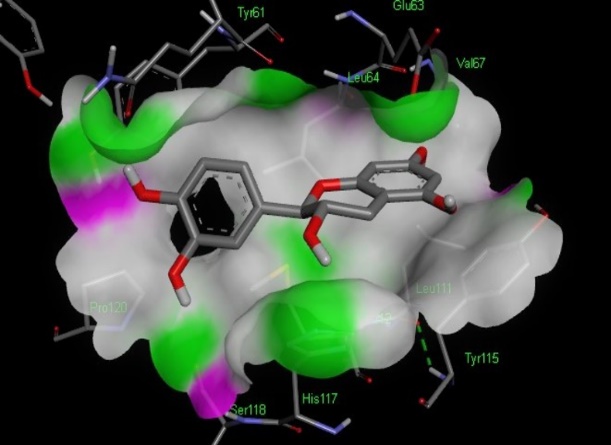 |  |
| --- | --- | --- |

**Fig S7:** Interaction **(A)** and binding pattern **(B)** of Epicatechin with SFRP4 Protein.

| **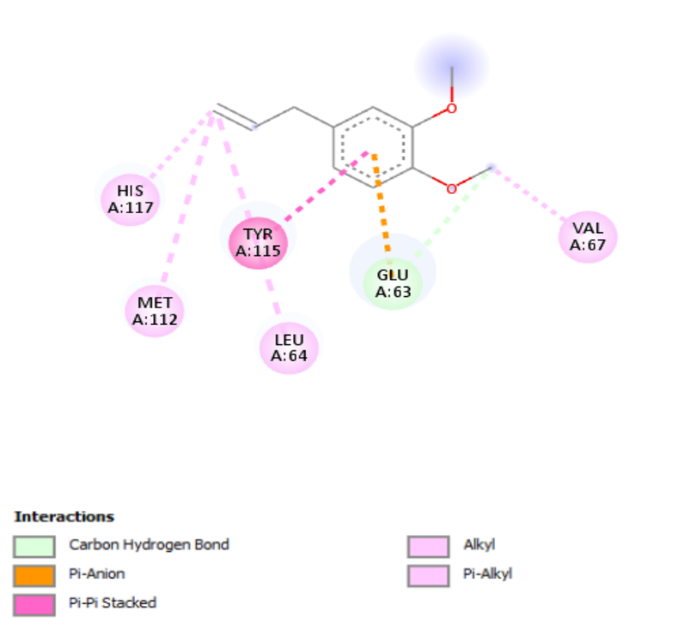**  **(B)**  **(A)** | 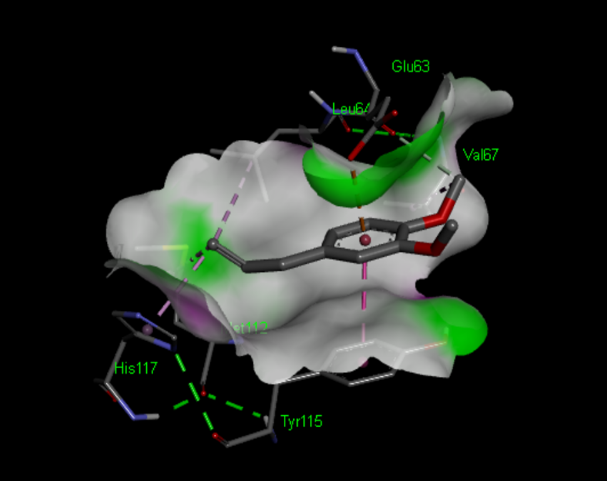 |  |
| --- | --- | --- |

**Fig S8:** Interaction **(A)** and binding pattern **(B)** of Methyl Eugenol with SFRP4 Protein.


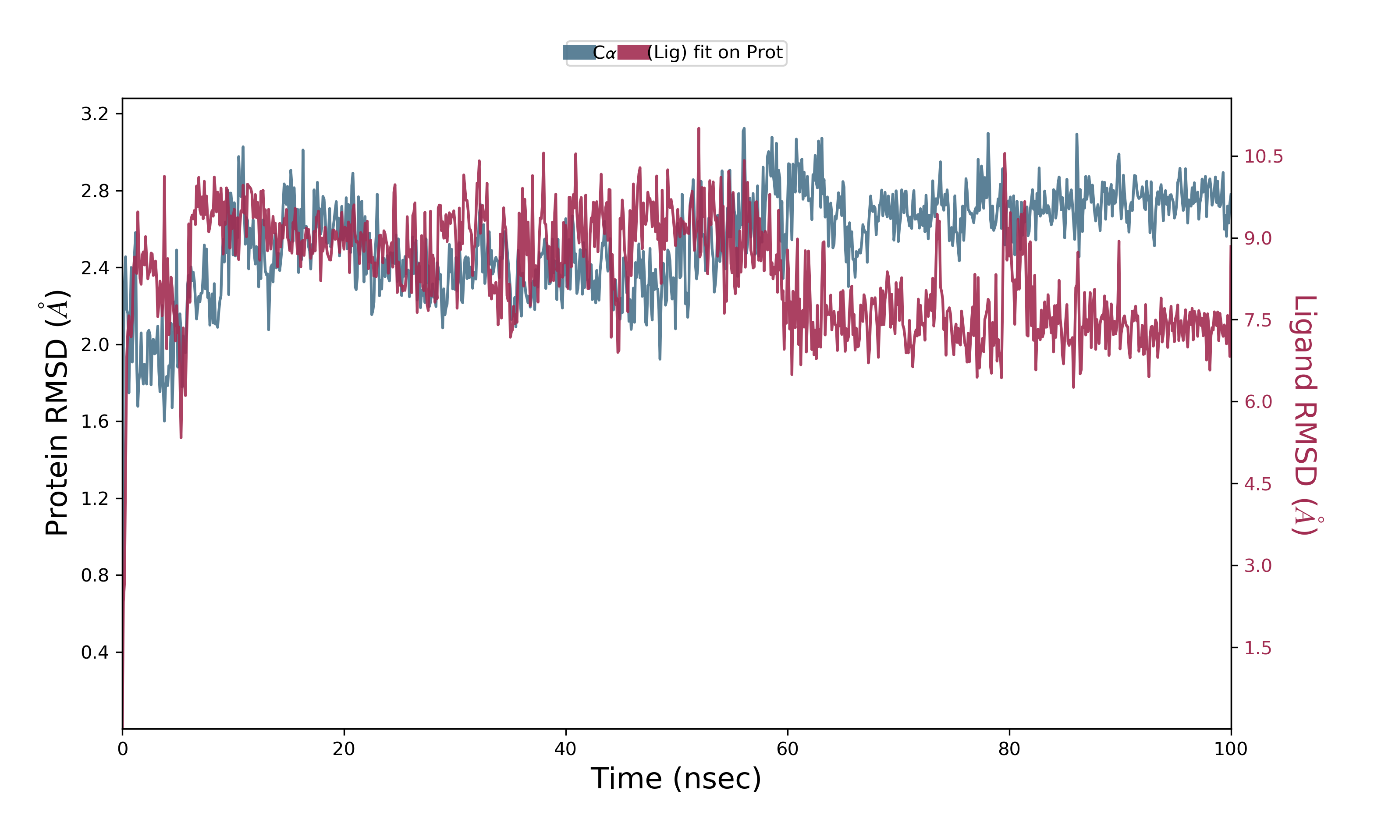


**Fig S9:** PL-RMSD graph Representation of Hesperetin with SFRP4


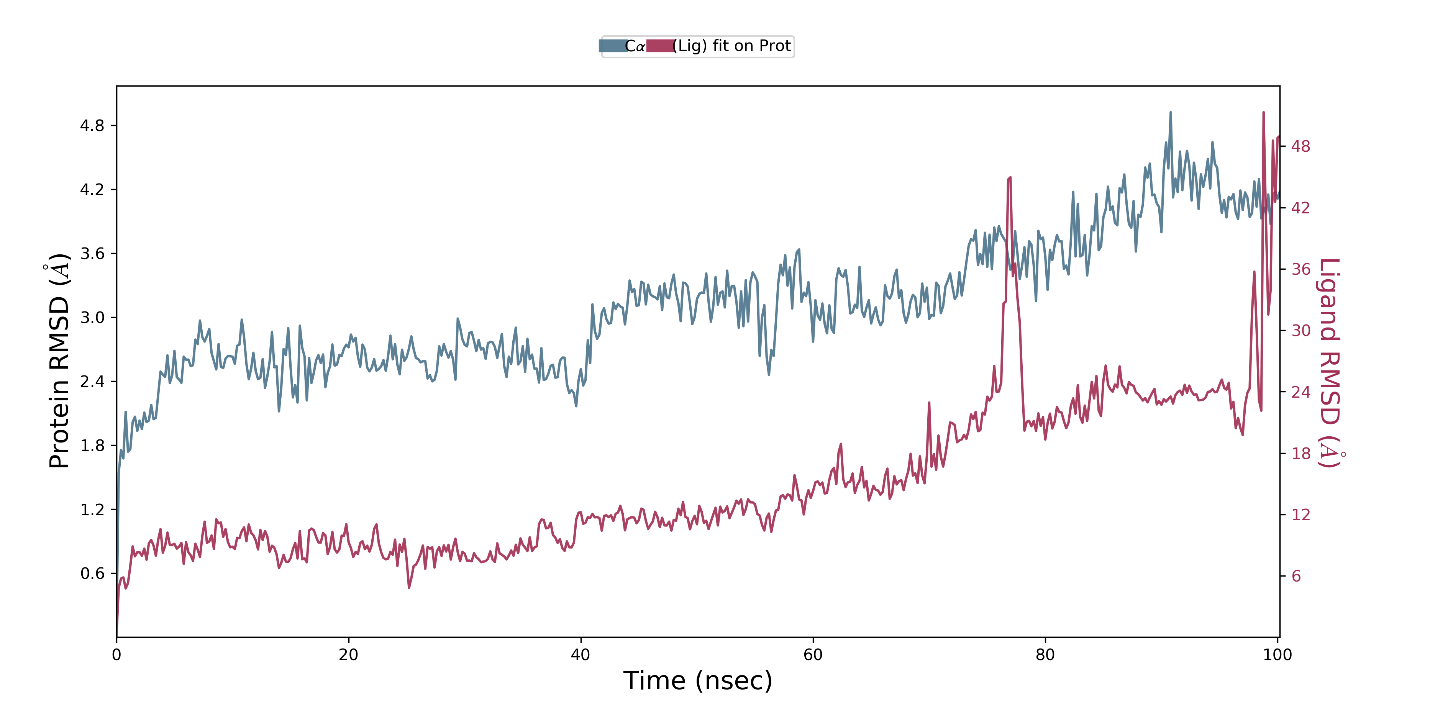


**Fig S10:** PL-RMSD graph Representation of Curcumin with SFRP4


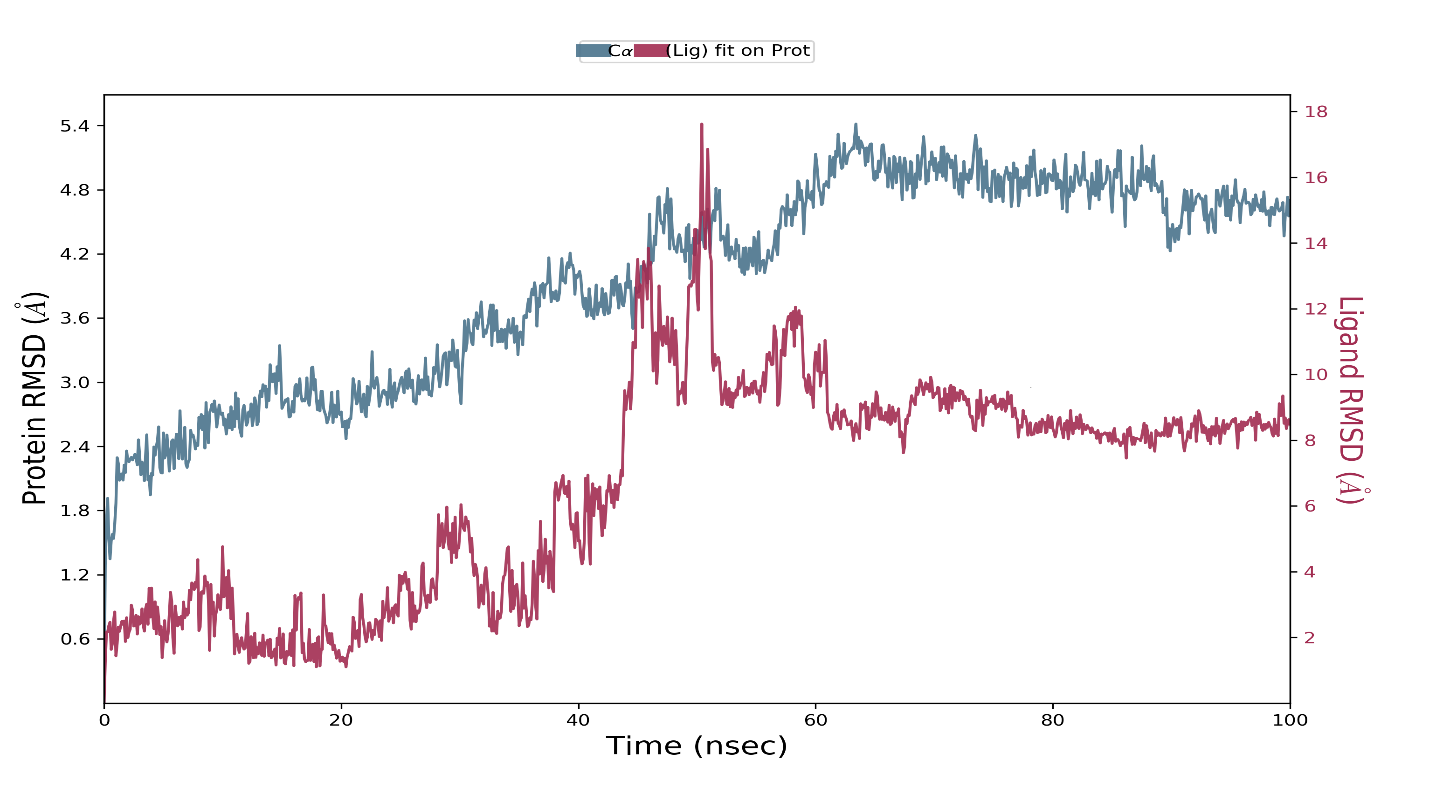


**Fig S11:** PL-RMSD graph Representation of Isorhamnetin with SFRP4


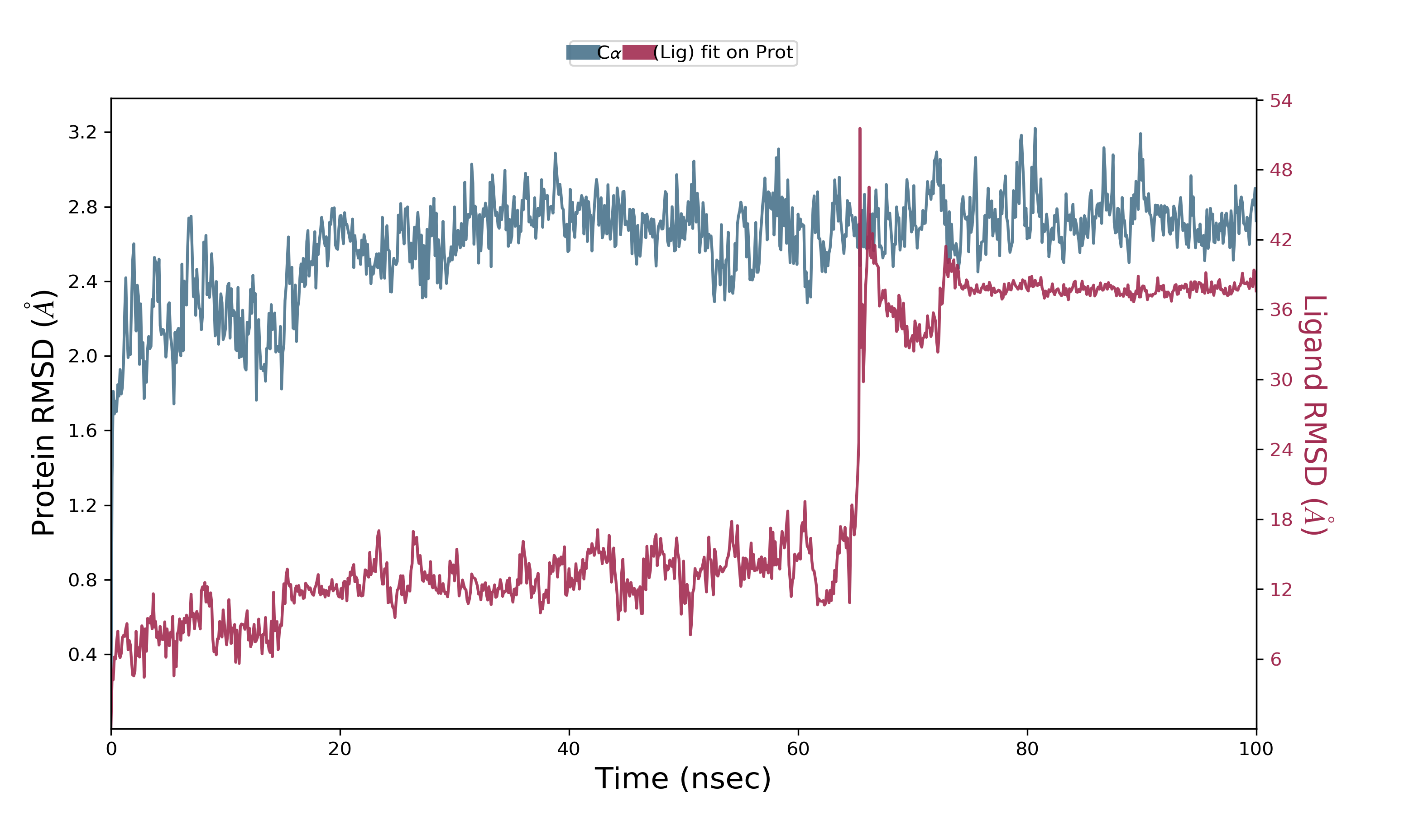


**Fig S12:** PL-RMSD graph Representation of Embelin with SFRP4


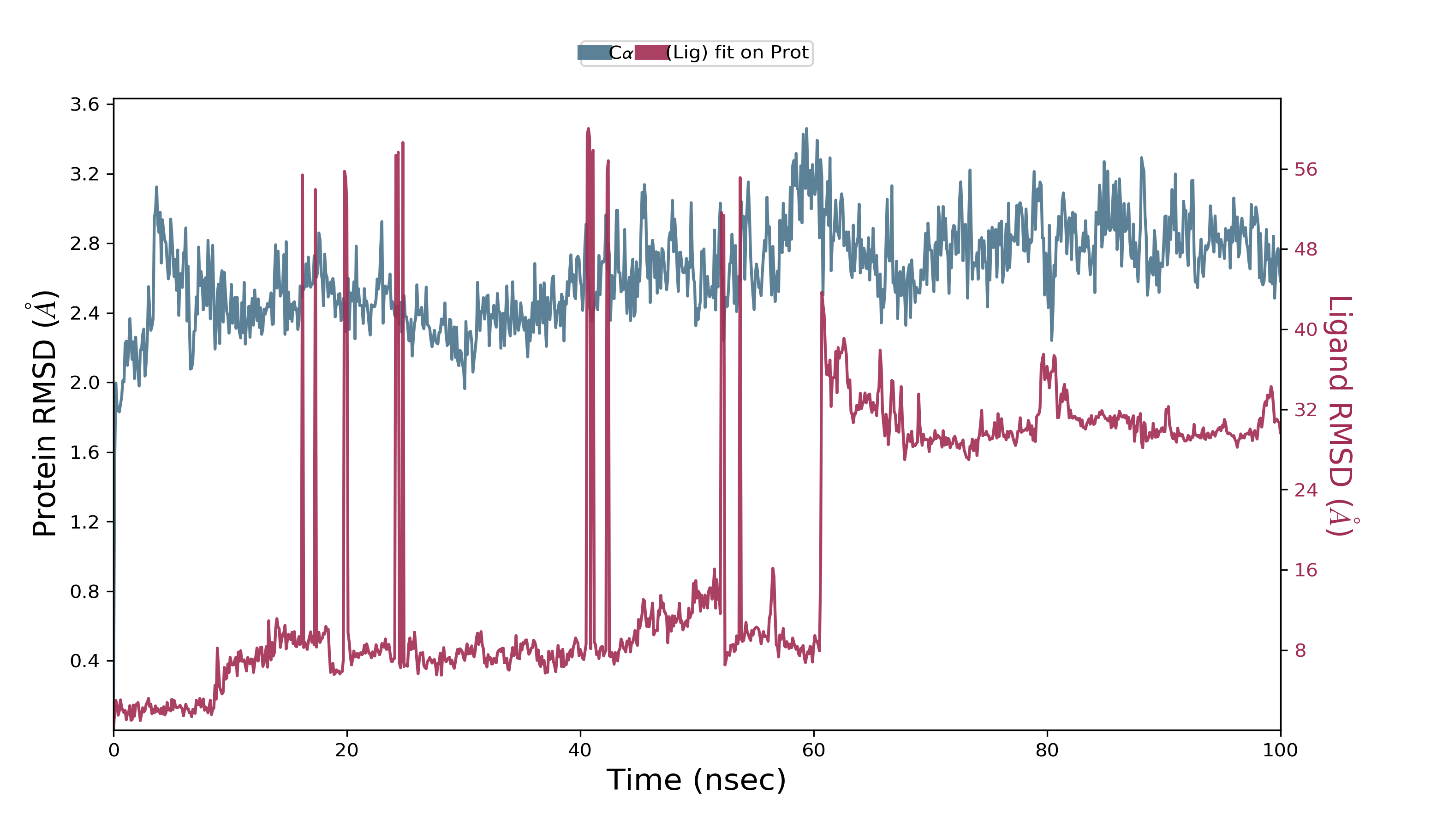


**Fig S13**: PL-RMSD graph Representation of Epicatechin with SFRP4


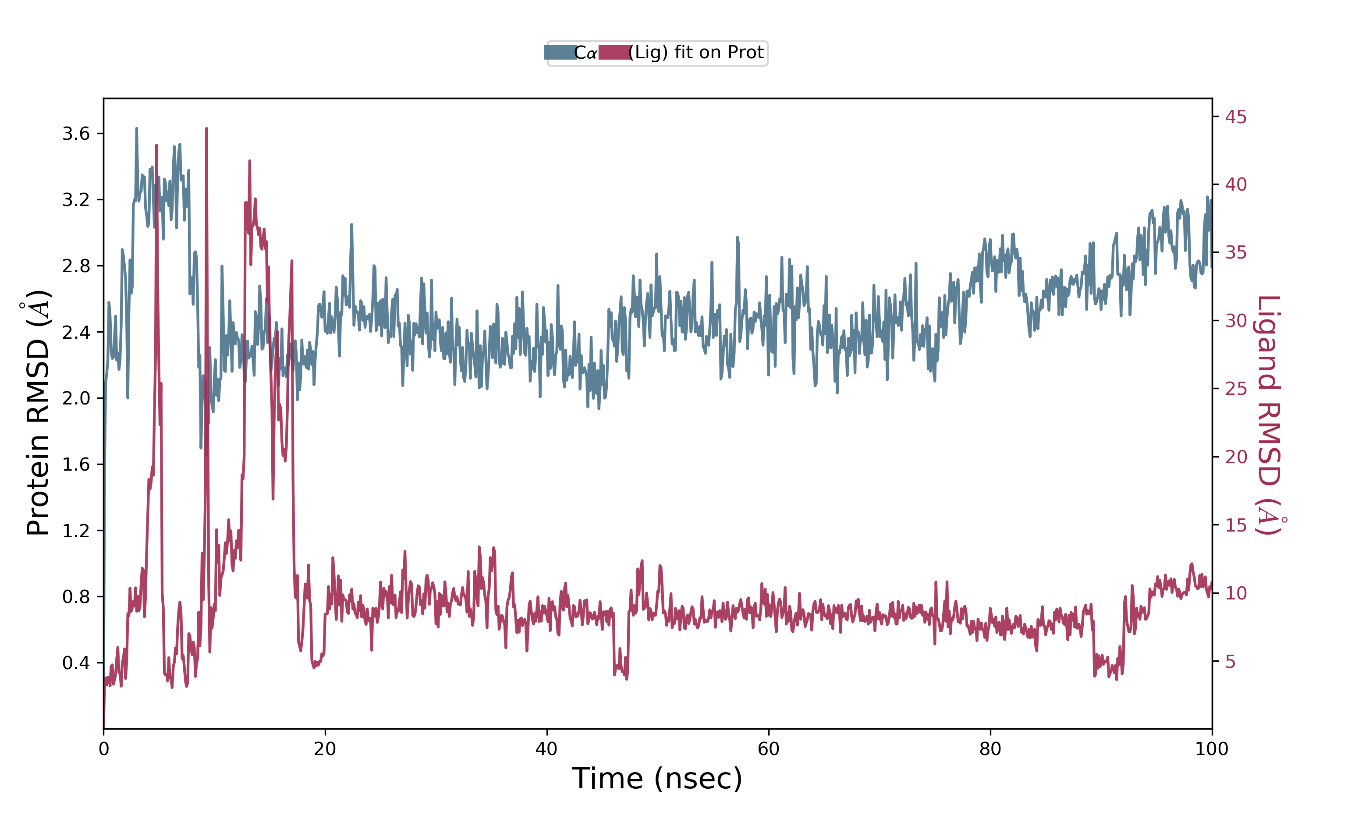


**Fig S14:** PL-RMSD graph Representation of Methyl eugenol with SFRP4


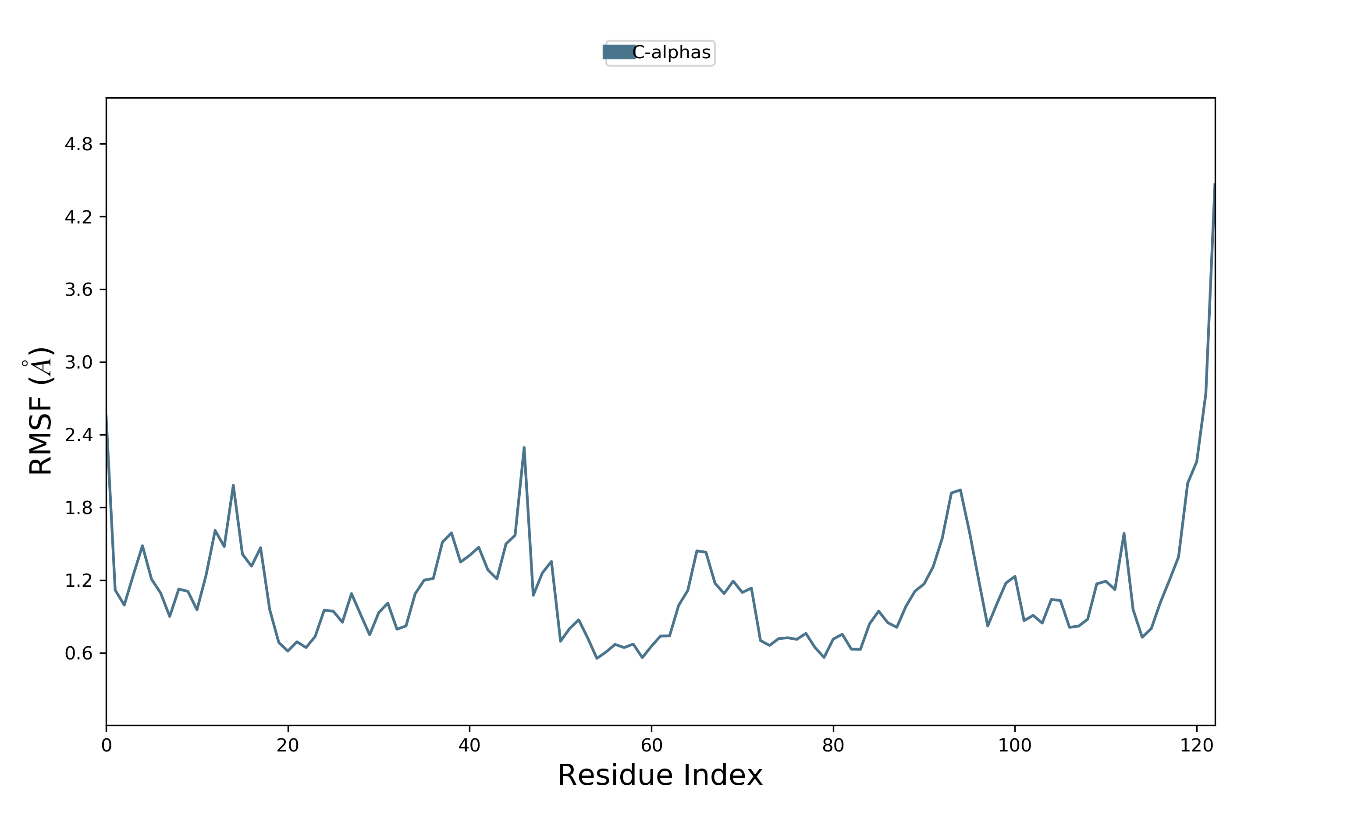


**Fig S15:** P-RMSF graph Representation of Hesperetin with SFRP4


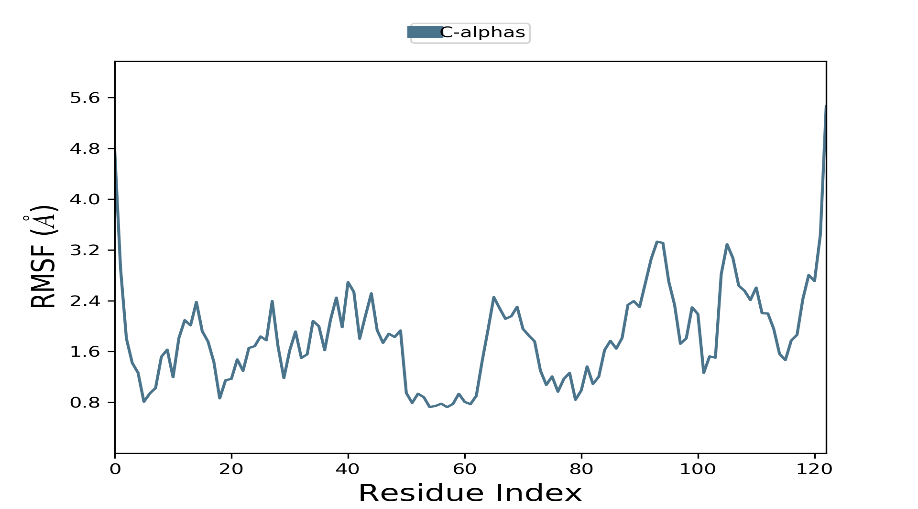


**Fig S16:** P-RMSF graph Representation of Curcumin with SFRP4


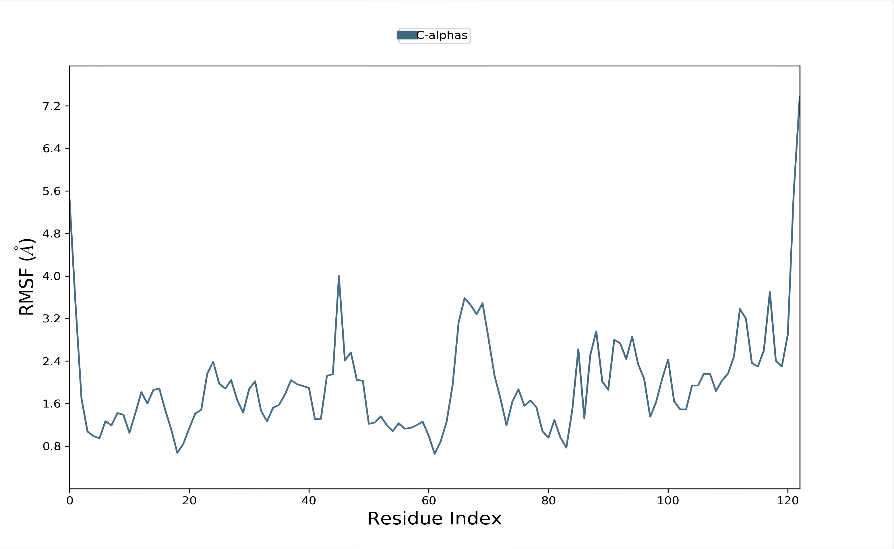


**Fig S17:** P-RMSF graph Representation of Isorhamnetin with SFRP4


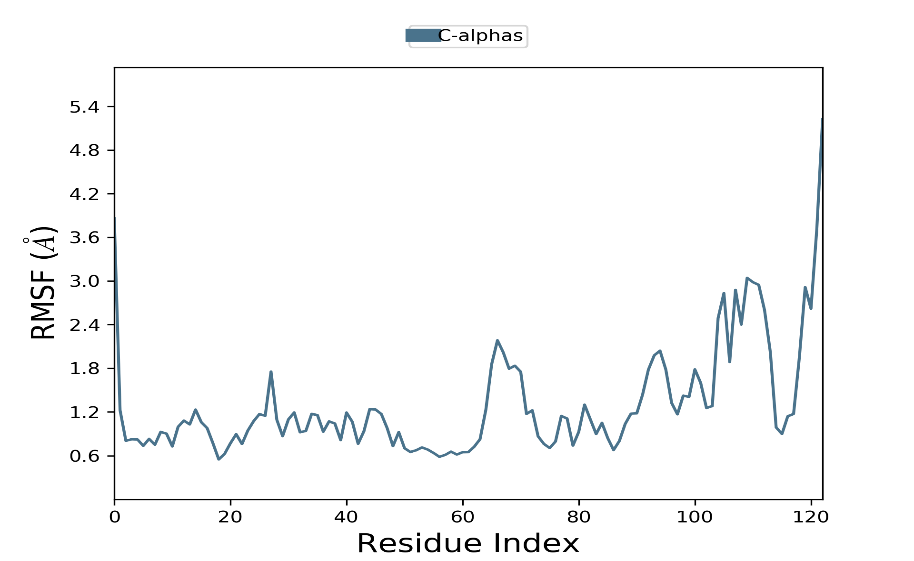


**Fig S18:** P-RMSF graph Representation of Embelin with SFRP4


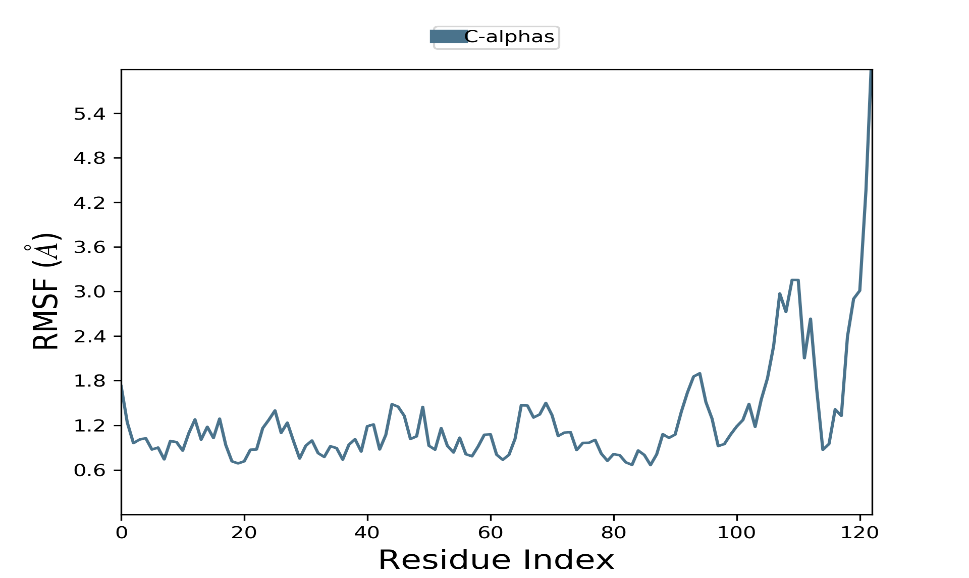


**Fig S19:** P-RMSF graph Representation of Epicatechin with SFRP4


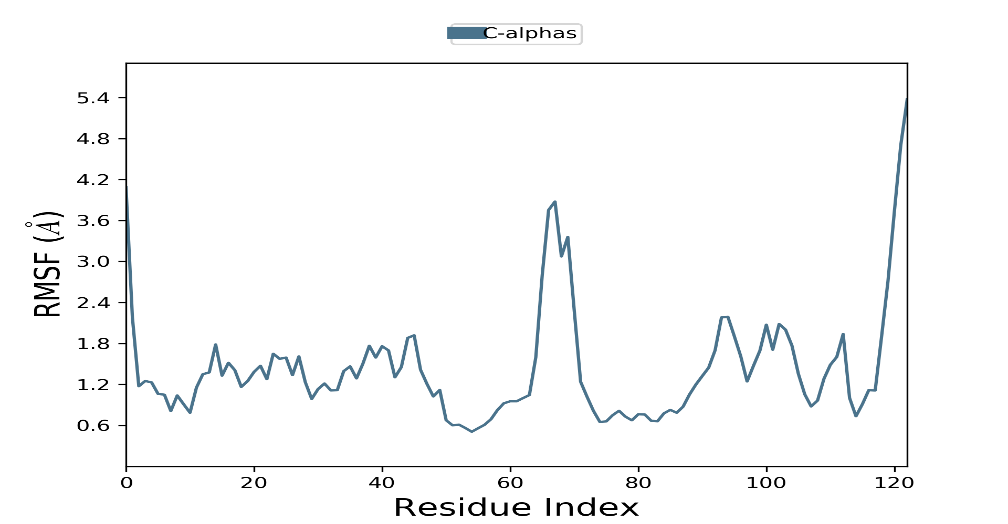


**Fig S20:** P-RMSF graph Representation of Methyl Eugenol with SFRP4


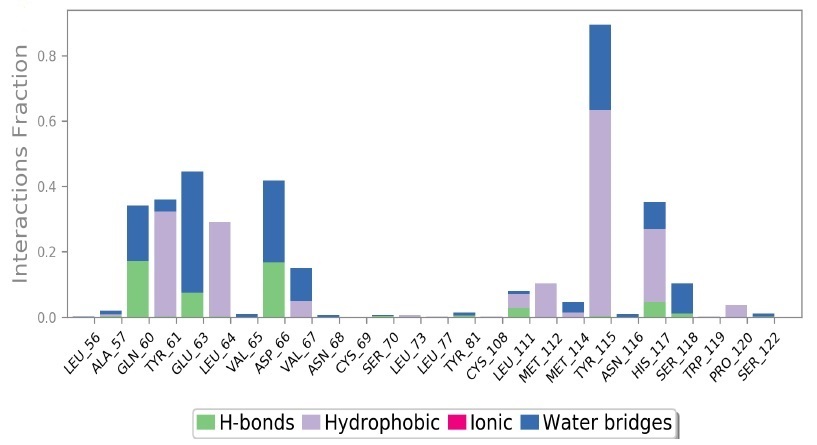


**Fig S21:** Protein-ligand interaction graph Representation of Hesperetin complex


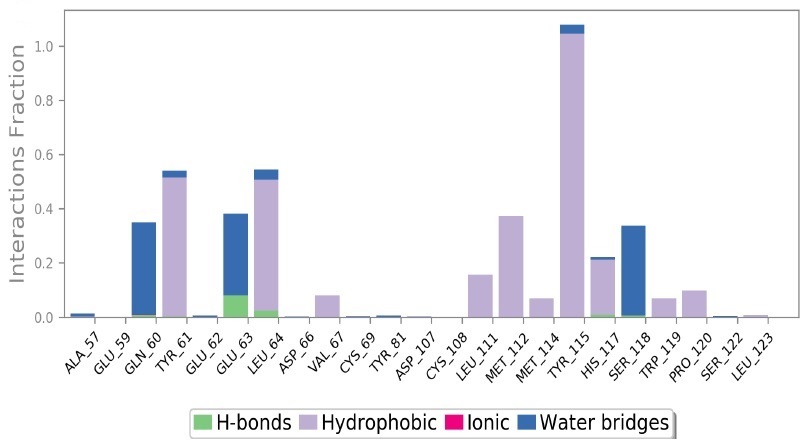


**Fig S22:** Protein-ligand interaction graph Representation of Curcumin complex

**
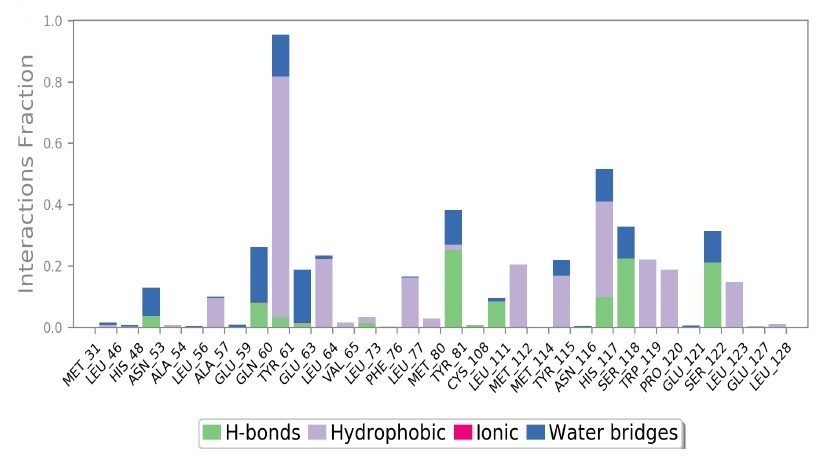
**

**Fig S23:** Protein-ligand interaction graph Representation of Isorhamnetin complex


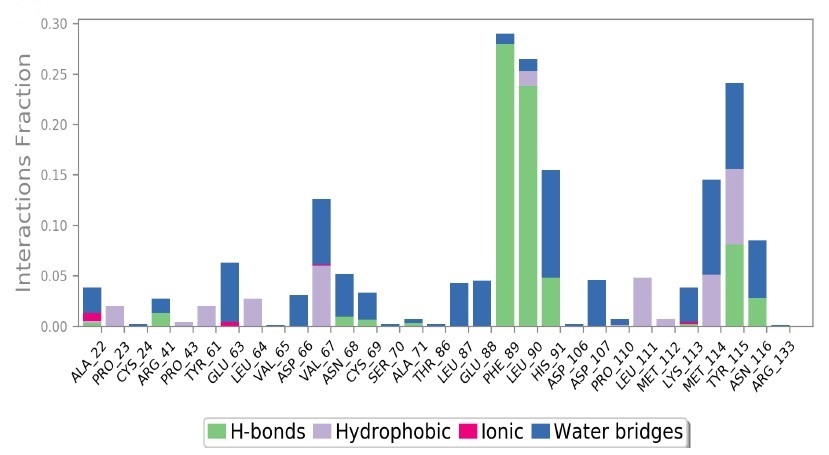


**Fig S24:** Protein-ligand interaction graph Representation of Embelin complex


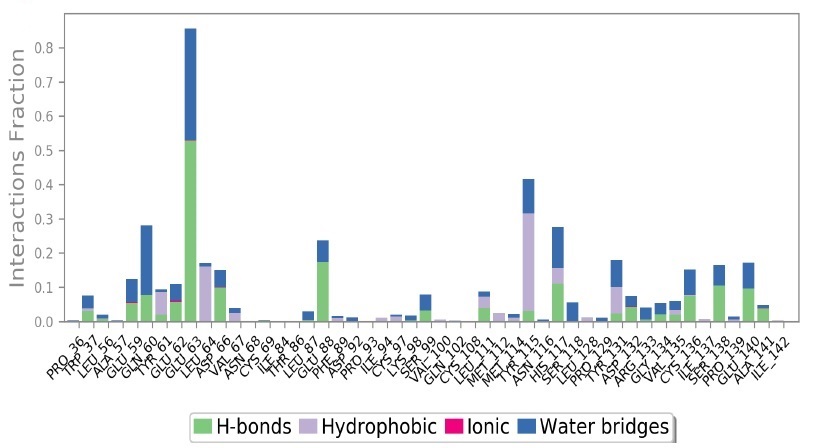


**Fig S25:** Protein-ligand interaction graph Representation of Epicatechin complex


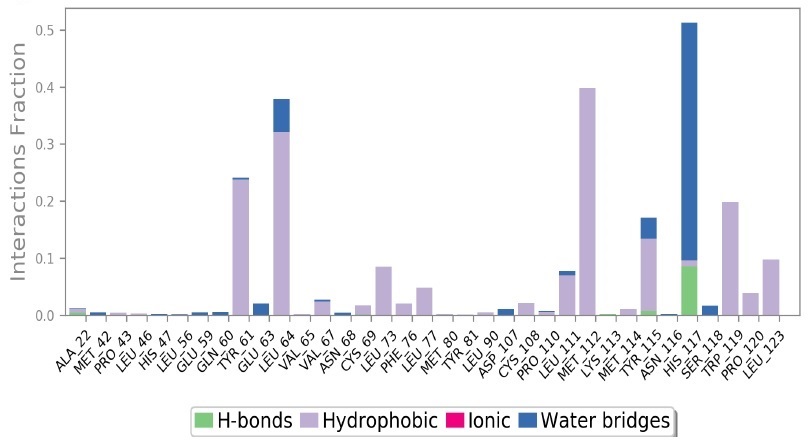


**Fig S26:** Protein-ligand interaction graph Representation of methyl eugenol complex

**A)**

**
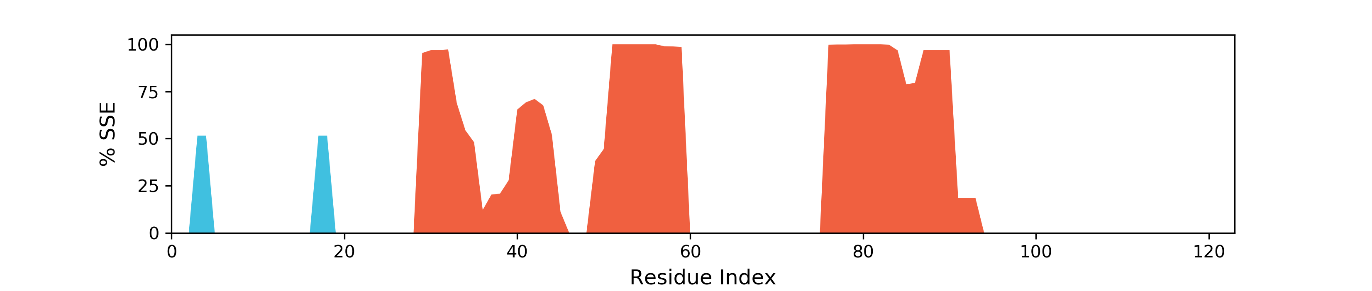
**

**B)**

**
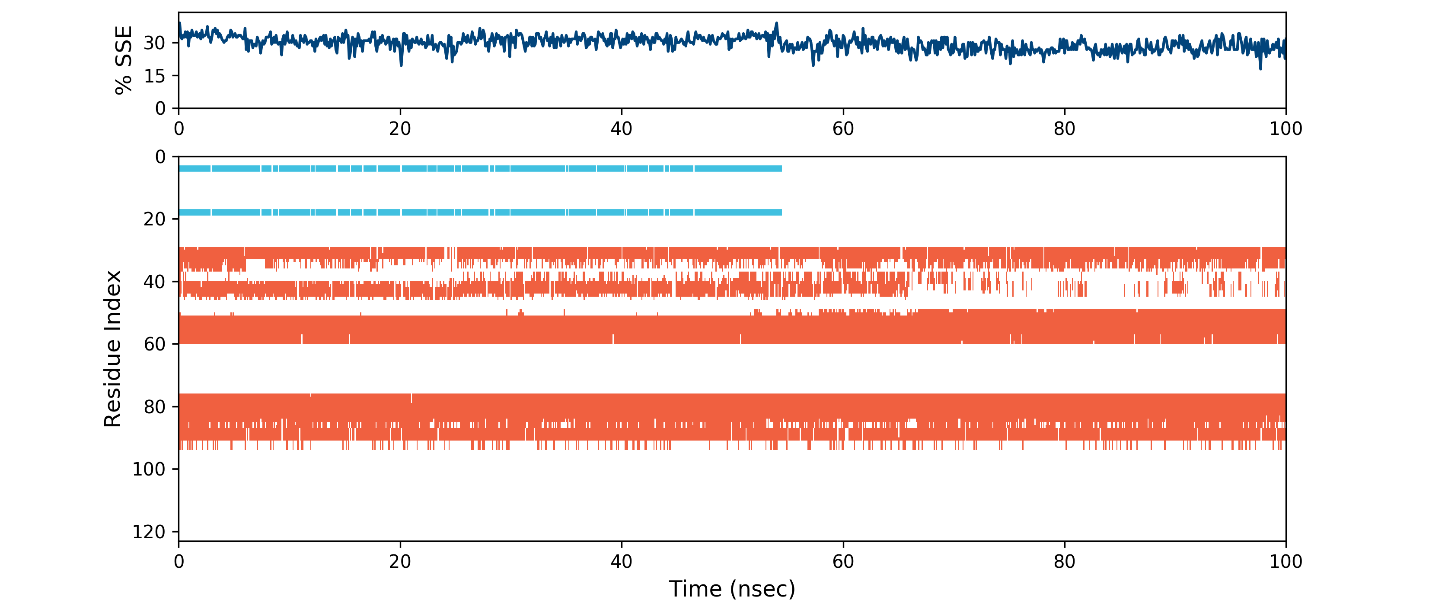
**

**Fig S27:** Protein Secondary Structure Elements of Hesperetin complex with receptor protein **(A)** Histogram and **(B)** Timeline throughout the Simulation

**A)**

**
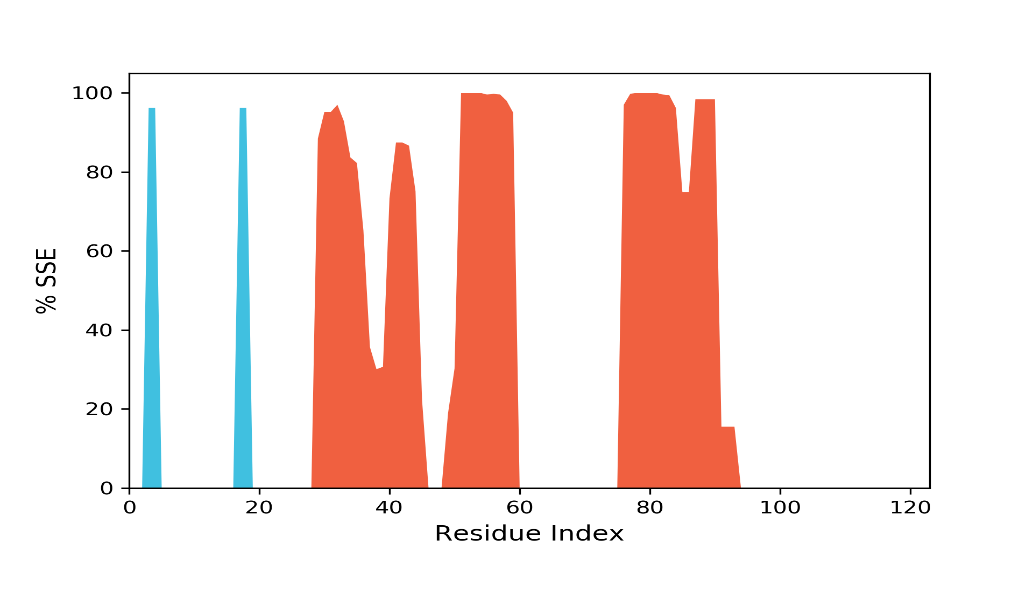
**

**B)
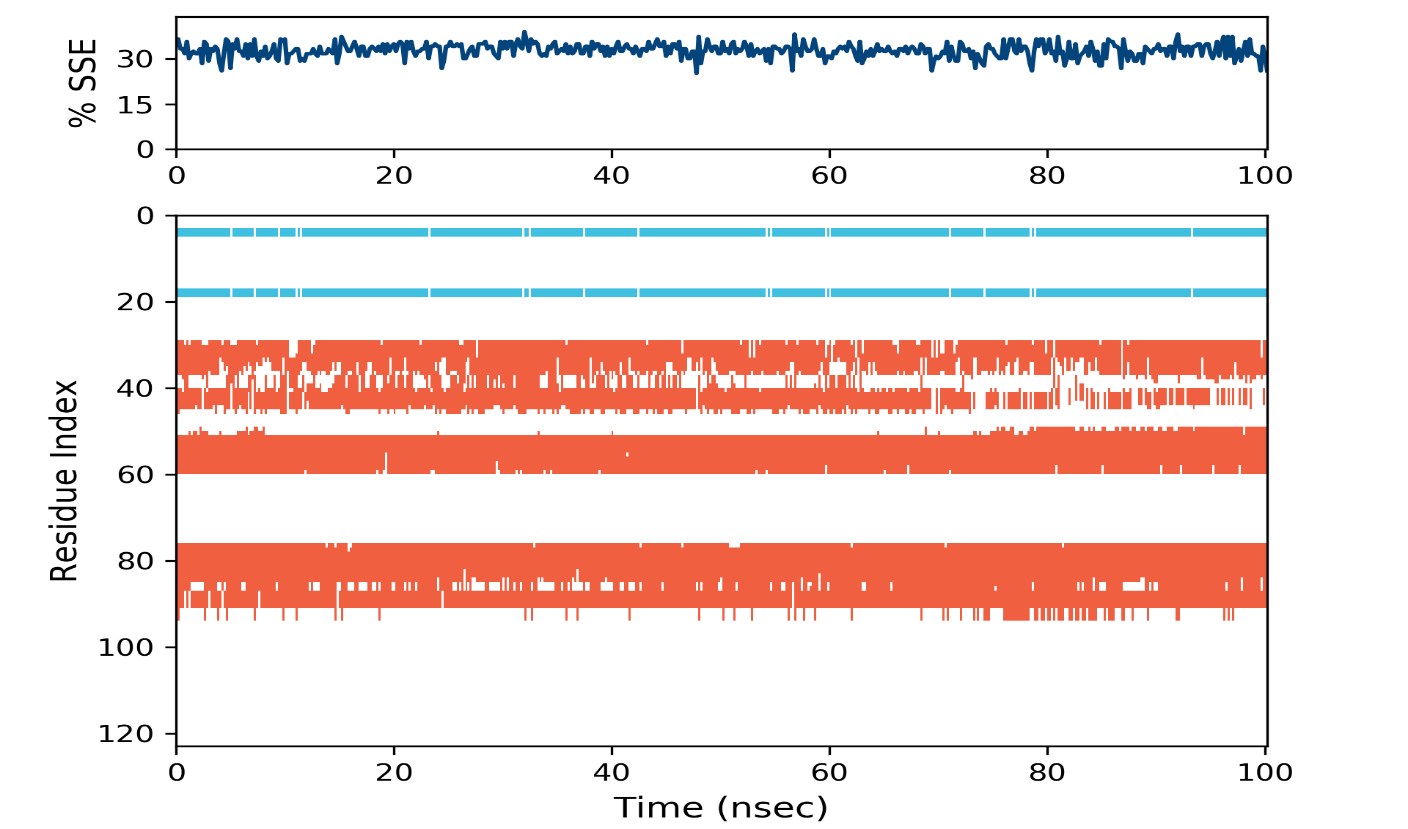
**

**Fig S28:** Protein Secondary Structure Elements of Curcumin complex with receptor protein **(A)** Histogram and **(B)** Timeline throughout the Simulation

**A)**

**
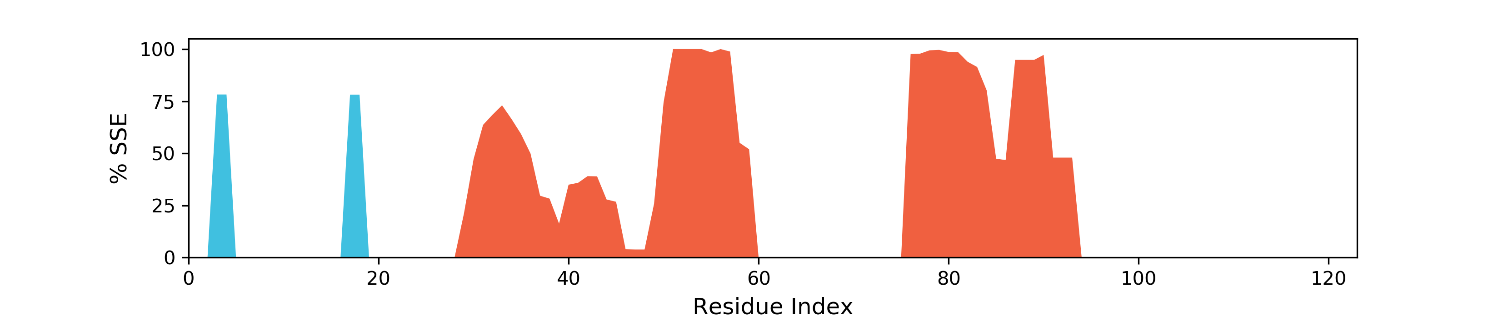
**

**B)
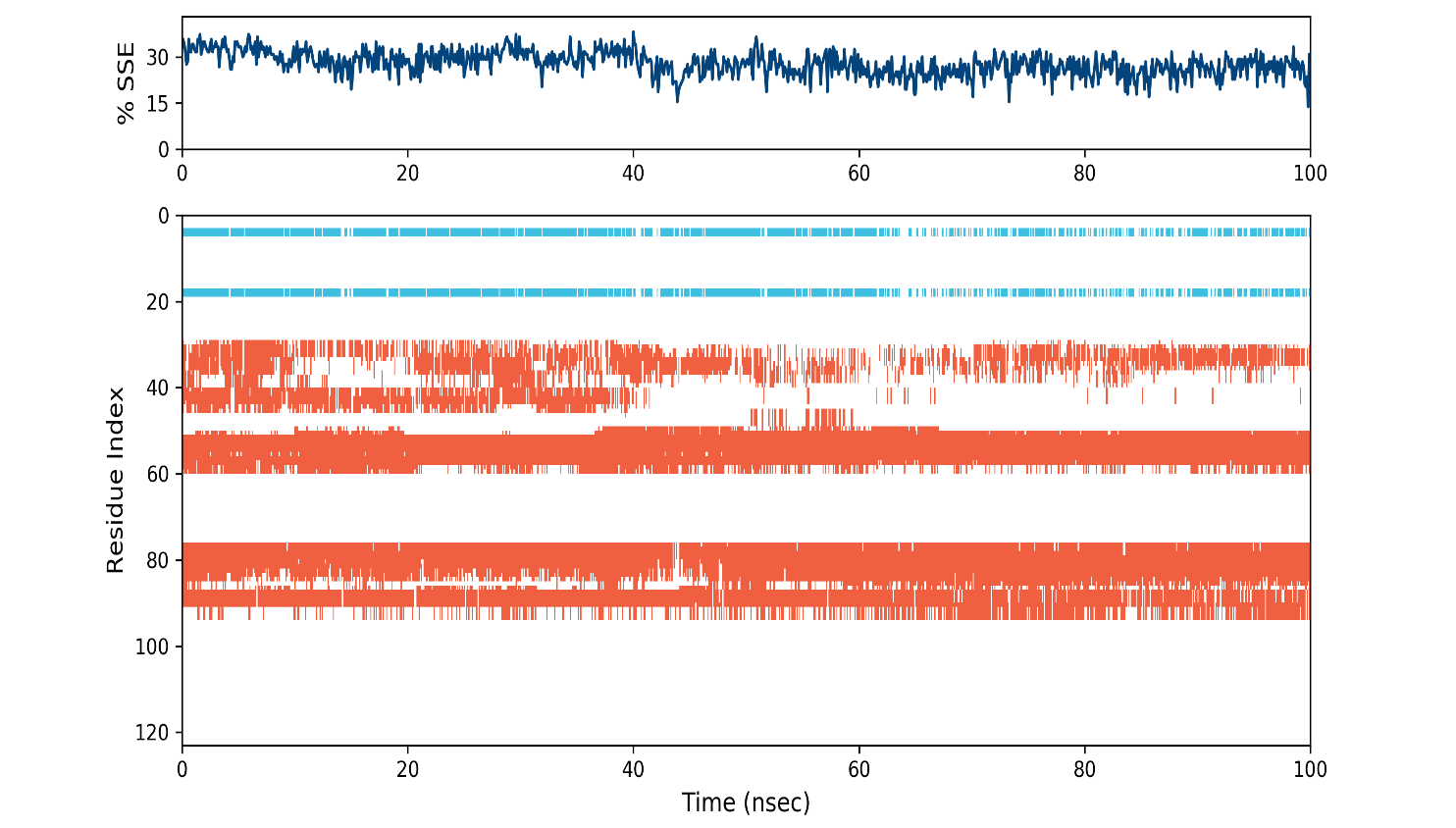
**

**Fig S29:** Protein Secondary Structure Elements of Isorhamnetin complex with receptor protein **(A)** Histogram and **(B)** Timeline throughout the Simulation

**A)**

**
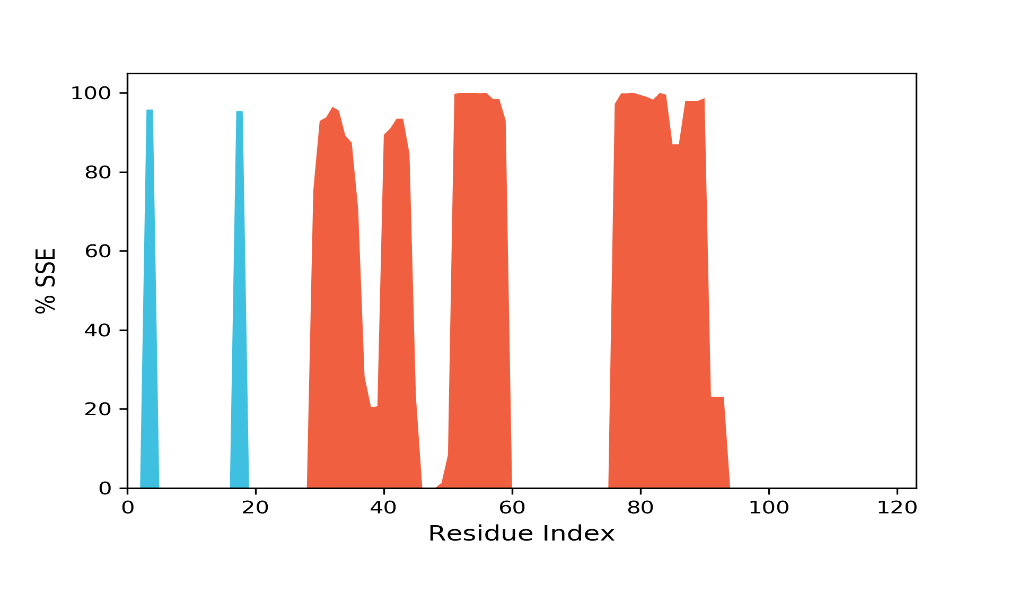
**

**B)**

**
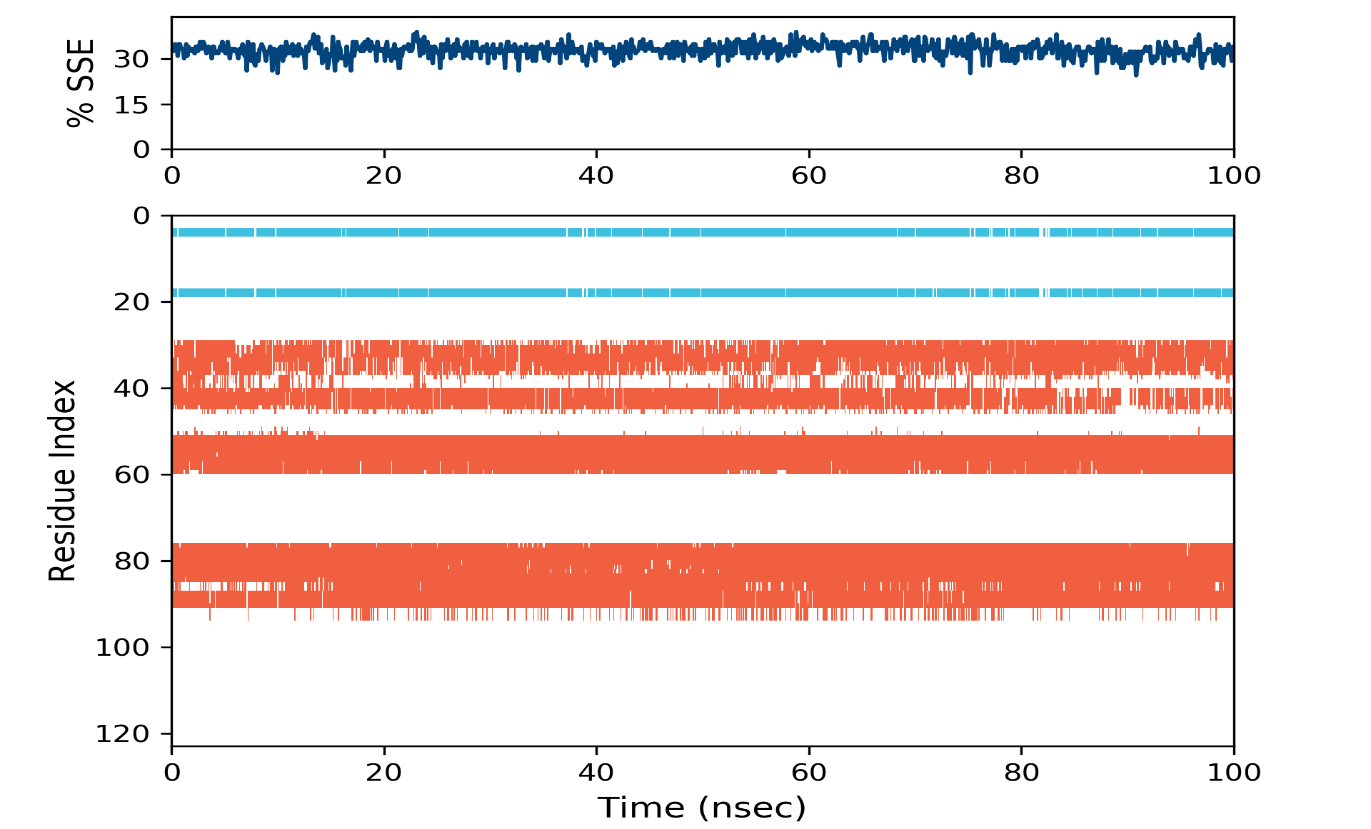
**

**Fig S30:** Protein Secondary Structure Elements of Embelin complex with receptor protein **(A)** Histogram and **(B)** Timeline throughout the Simulation

**A)**

**
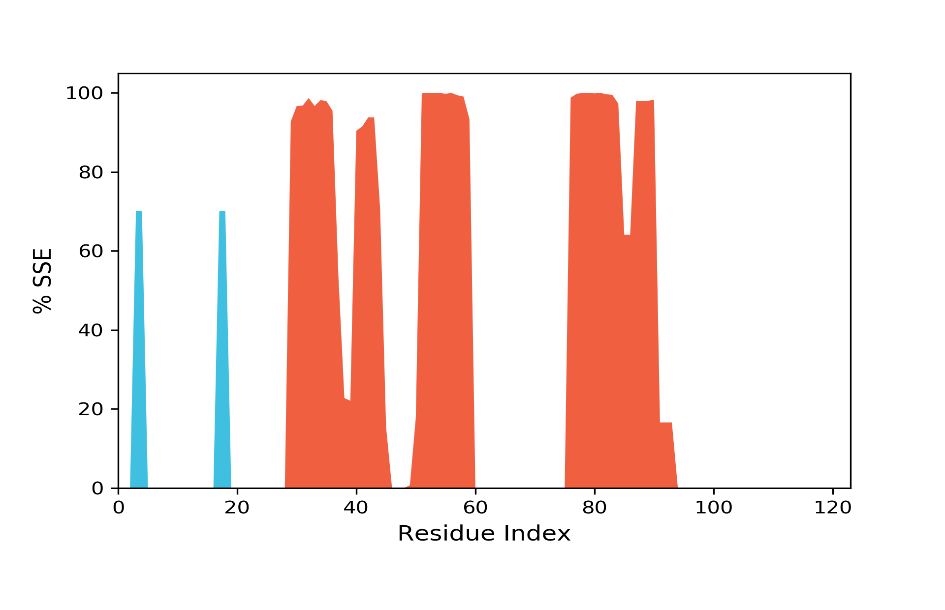
**

**B)**

**
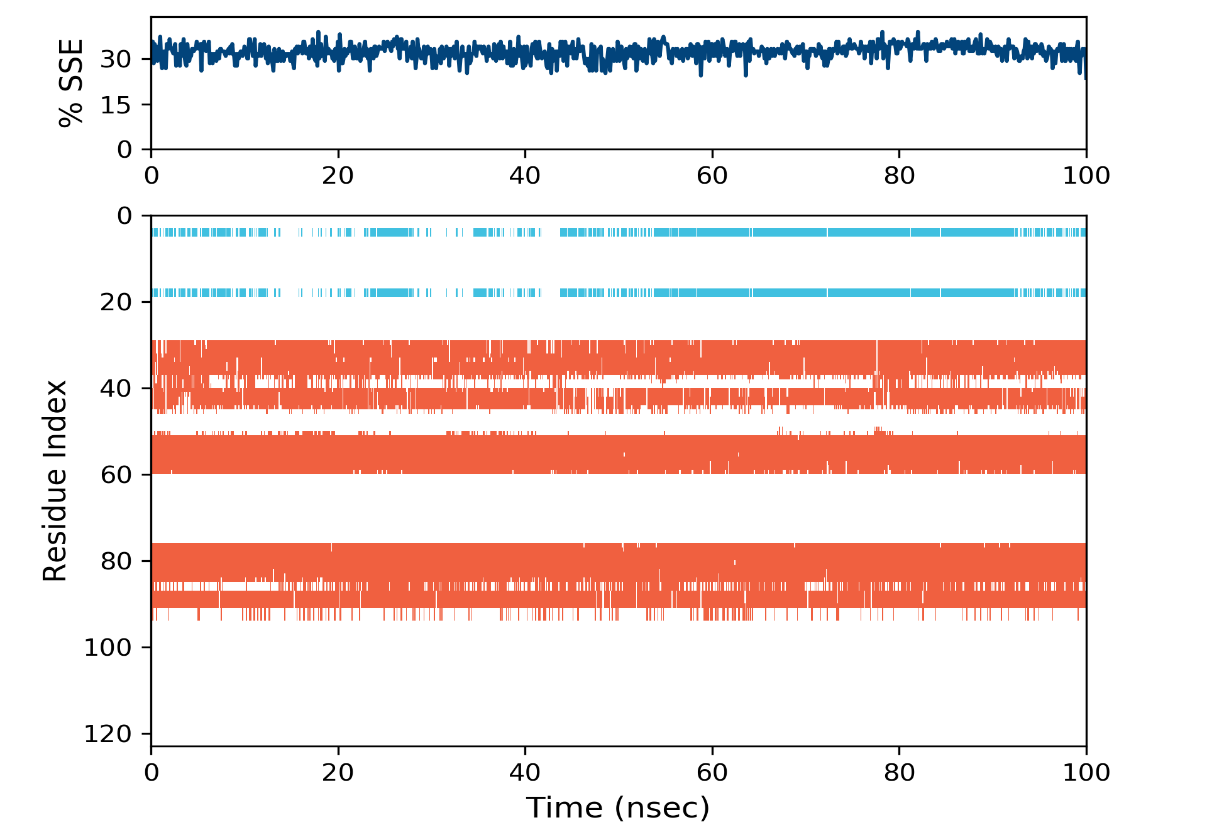
**

**Fig S31:** Protein Secondary Structure Elements of Epicatechin complex with receptor protein **(A)** Histogram and **(B)** Timeline throughout the Simulation

**A)**

**
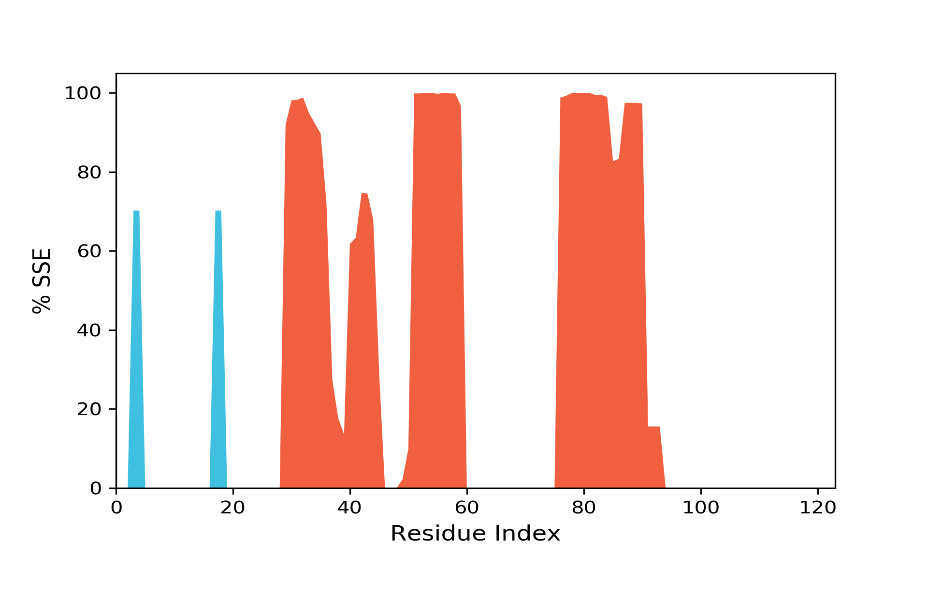
**

**B)**

**
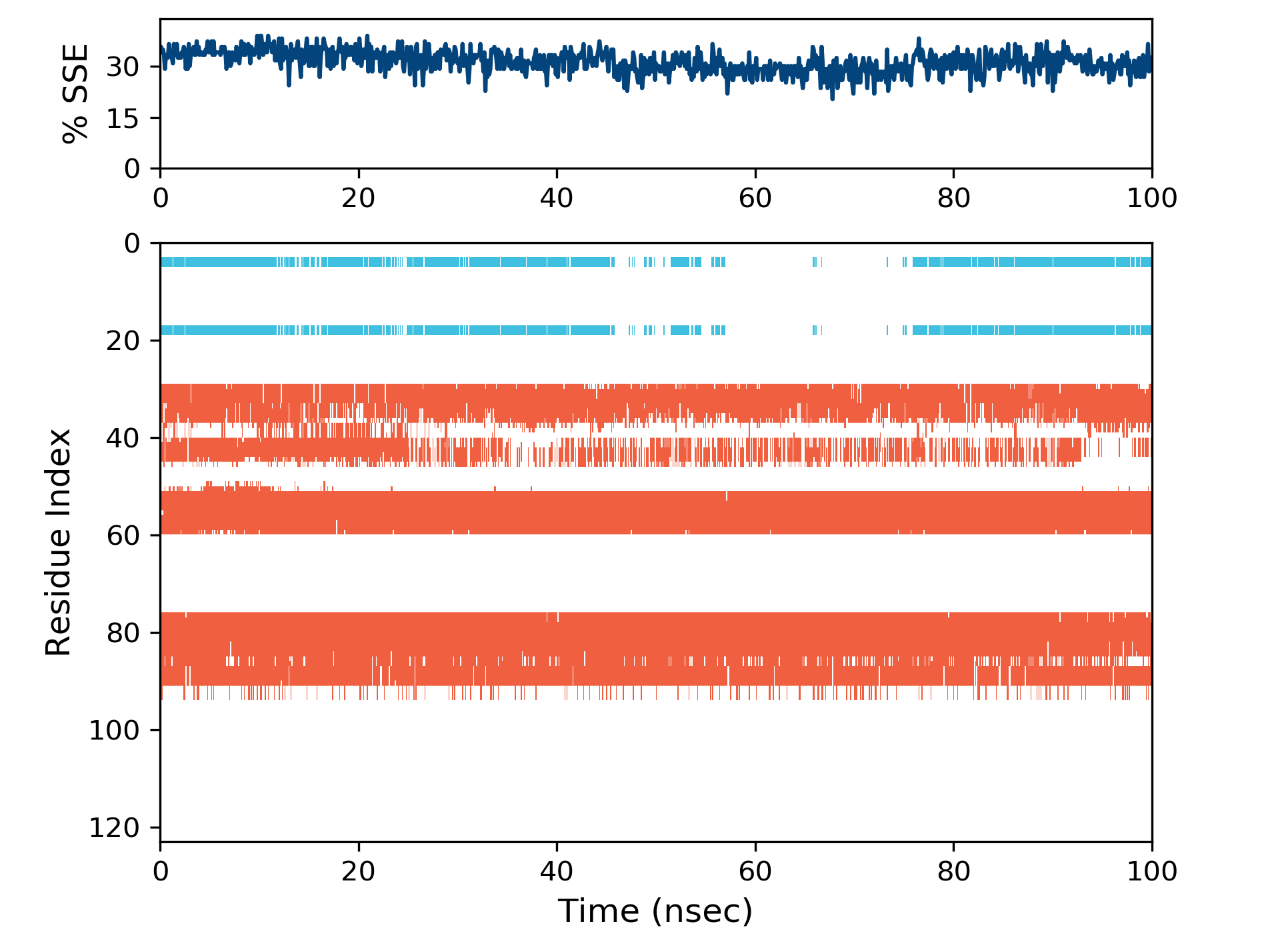
**

**Fig S32:** Protein Secondary Structure Elements of Methyl eugenol complex with receptor protein **(A)** Histogram and **(B)** Timeline throughout the Simulation
